# Large-Scale Identification of MicroRNA Targets in Murine *Dgcr8*-Deficient Embryonic Stem Cell Lines

**DOI:** 10.1371/journal.pone.0041762

**Published:** 2012-08-17

**Authors:** Matthew P. A. Davis, Cei Abreu-Goodger, Stijn van Dongen, Dong Lu, Peri H. Tate, Nenad Bartonicek, Claudia Kutter, Pentao Liu, William C. Skarnes, Anton J. Enright, Ian Dunham

**Affiliations:** 1 Wellcome Trust Sanger Institute, Wellcome Trust Genome Campus, Hinxton, United Kingdom; 2 European Bioinformatics Institute, European Molecular Biology Laboratory, Wellcome Trust Genome Campus, Hinxton, United Kingdom; 3 National Laboratory of Genomics for Biodiversity (Langebio), Cinvestav, Irapuato, Guanajuato, Mexico; 4 Cancer Research UK, Cambridge Research Institute, Li Ka Shing Centre, Cambridge, United Kingdom; Hemocentro de Ribeirão Preto, HC-FMRP-USP., Brazil

## Abstract

Small RNAs such as microRNAs play important roles in embryonic stem cell maintenance and differentiation. A broad range of microRNAs is expressed in embryonic stem cells while only a fraction of their targets have been identified. We have performed large-scale identification of embryonic stem cell microRNA targets using a murine embryonic stem cell line deficient in the expression of *Dgcr8*. These cells are heavily depleted for microRNAs, allowing us to reintroduce specific microRNA duplexes and identify refined target sets. We used deep sequencing of small RNAs, mRNA expression profiling and bioinformatics analysis of microRNA seed matches in 3′ UTRs to identify target transcripts. Consequently, we have identified a network of microRNAs that converge on the regulation of several important cellular pathways. Additionally, our experiments have revealed a novel candidate for *Dgcr8*-independent microRNA genesis and highlighted the challenges currently facing miRNA annotation.

## Introduction

MicroRNAs (miRNAs) are important mediators of post-transcriptional gene regulation. They are RNA molecules ∼22 nt in length, responsible for guiding the RNA induced silencing complex (RISC) to mRNA molecules, predominantly through complementarity between the 5′ end of the miRNA (containing the seed region) and sequences within the 3′ UTR of the target molecules. This can lead to degradation of the targeted mRNA and inhibition of its translation. There are examples of these mechanisms acting independently, but it has recently become clear that in the majority of cases a miRNAs will reduce both protein and mRNA levels of a target [1]–[3]. A large proportion of the cellular transcriptome is thought to be regulated by miRNAs, with over 60% of human genes predicted to be the conserved targets of one or more miRNA [4].

Transcribed within much larger RNA sequences (pri-miRNA), miRNAs are released by series of RNase III processing reactions. In the nucleus the precursor miRNA (pre-miRNA) hairpin is released by the RNase III enzyme DROSHA, operating in conjunction with the double stranded RNA binding protein, DiGeorge syndrome critical region gene 8 (DGCR8) [5]–[9]. Both components of this microprocessor complex are required for canonical miRNA processing [6], [7]. Subsequently the pre-miRNA hairpin is exported to the cytoplasm where Dicer (DICER1) is responsible for releasing the miRNA from this hairpin [10]–[12]. Finally, the miRNA is incorporated into the microRNA-induced silencing complex (miRISC).

The number of annotated miRNAs has expanded enormously over the course of the last decade. Currently there are 741 mouse-miRNA hairpins annotated in miRBase (release 18) [13]. However, despite well-established systems to identify new miRNAs, it has proven difficult to annotate the rapidly growing list of miRNAs with individual functions. Although efforts are being made to experimentally identify large numbers of miRNA targets [14], [15], to date computational target prediction remains one of the most widely used tools for the generation of hypotheses regarding miRNA function and potential miRNA:target interactions [4], [16], [17]. However, given the scale of the problem of functional annotation, many of these predictions are yet to incorporate *in vitro* or *in vivo* conditions that may influence target selection, such as the co-expression of targets and miRNAs.

Embryonic stem (ES) cells, which are derived from the inner cell mass of the blastocyst, are capable of self-renewal and are pluripotent, capable of differentiating into all somatic lineages. As such they provide an *in vitro* model for development and a system that possesses considerable therapeutic potential. Recently, several systems have been developed that provide an insight into the roles that miRNAs play in ES cells by knocking out components of the miRNA processing pathway [18]–[20]. Depletion of both DICER1 and DGCR8 proteins in mouse ES cells perturbs the cell cycle leading to an accumulation of cells in the G1 phase [18], [19]. These mutant ES cells are also unable to complete differentiation. *Dicer1* knockout cells maintain ES cell marker expression [20] and *Dgcr8* knockout cells are able to revert to an undifferentiated state once the differentiation conditions are reversed [18]. By studying systems such as these it has become apparent that one of the most highly expressed mouse ES cell miRNA clusters (the miR-290 cluster [21]) plays a fundamental role in the regulation of the mouse ES cell cell-cycle and differentiation [22], [23]. Many of the miRNAs within this cluster and other miRNAs that are highly expressed in mouse ES cells, share a high degree of sequence identity within their seed region and are consequently expected to share target mRNAs. Indeed these miRNAs have demonstrated a degree of functional redundancy in their regulation of the embryonic stem cell cycle [22].

We describe a mouse ES cell line depleted in the expression of *Dgcr8* and canonically processed miRNAs. This allows us to reintroduce miRNAs into a system with limited miRNA functional redundancy so targets should no longer be saturated by endogenous miRNA expression. Through a simple system by which miRNAs are reintroduced individually to these cells and subsequent mRNA expression changes are measured by microarray, we were able to partially rescue the wild-type ES cell mRNA expression profile and identify lists of mRNA transcripts that are likely targets of a number of miRNAs within wild type ES cells. In this way we are able to propose functions for individual miRNAs, uncover a broad network of the targets of miRNAs in ES cells and identify both basal transcription factors and the mediator complex as global/shared routes by which ES cell miRNAs appear to converge to regulate a wider cohort of secondary targets within these cells.

## Results

### Generation and validation of *Dgcr8*-deficient ES cell lines

In order to deplete mouse ES cells of miRNAs, both alleles of *Dgcr8* were disrupted. Targeted trapping of the second allele of *Dgcr8* was performed in two independent gene trap cell lines from the BayGenomics resource (Figure S1) [24]. RT-PCR was used to identify homozygous clones that contain both the trapped and targeted trapped alleles (data not shown). Two independently derived homozygous mutant cell lines were used in this study and are designated *Dgcr8^gt1/tm1^* and *Dgcr8^gt2/tm1^*. As controls, heterozygous cell lines were recovered, in the same electroporation, where the trapped allele was targeted in cis (*Dgcr8^tm1,gt1/+^* and *Dgcr8^tm1,gt2/+^*; Figure S2). Disruption of the locus was confirmed by RNA blot (Figure S3).

### Analysis of miRNA expression by small RNA sequencing

To investigate the functional consequences of disrupting the *Dgcr8* locus, small RNA libraries were prepared from the *Dgcr8^+/+^*, *Dgcr8^tm1,gt1/+^*, *Dgcr8^tm1,gt2/+^*, *Dgcr8^gt1/tm1^* and *Dgcr8^gt2/tm1^* cell lines and sequenced (see Materials and Methods). Each sequence library was then mapped to a database of sequences including all Ensembl annotated non-coding RNAs (ncRNAs) and the complete set of mouse miRNAs derived from miRBase [13] (Figure S4A). Mapped sequence coverage for each of the ncRNAs was used as a surrogate for its expression within each cell line. The maximum depth of coverage for each class of RNA was compared between the wild type (WT) cells and the 4 heterozygous and *Dgcr8*-deficient cell lines in a pairwise fashion (Figure S5). It is thought that a number of these RNA classes will be sequenced in a *Dgcr8*-independent manner [25] and this is supported by the consistent relative number of reads that map to the broad range of non-miRNAs across these contrasts. Considering this relationship, and in a fashion similar to that used by Babiarz *et al.* [25], the non-miRNA ncRNA species were used as the reference for the normalisation of each library using depth-scaling (see Materials and Methods).

**Figure 1:**
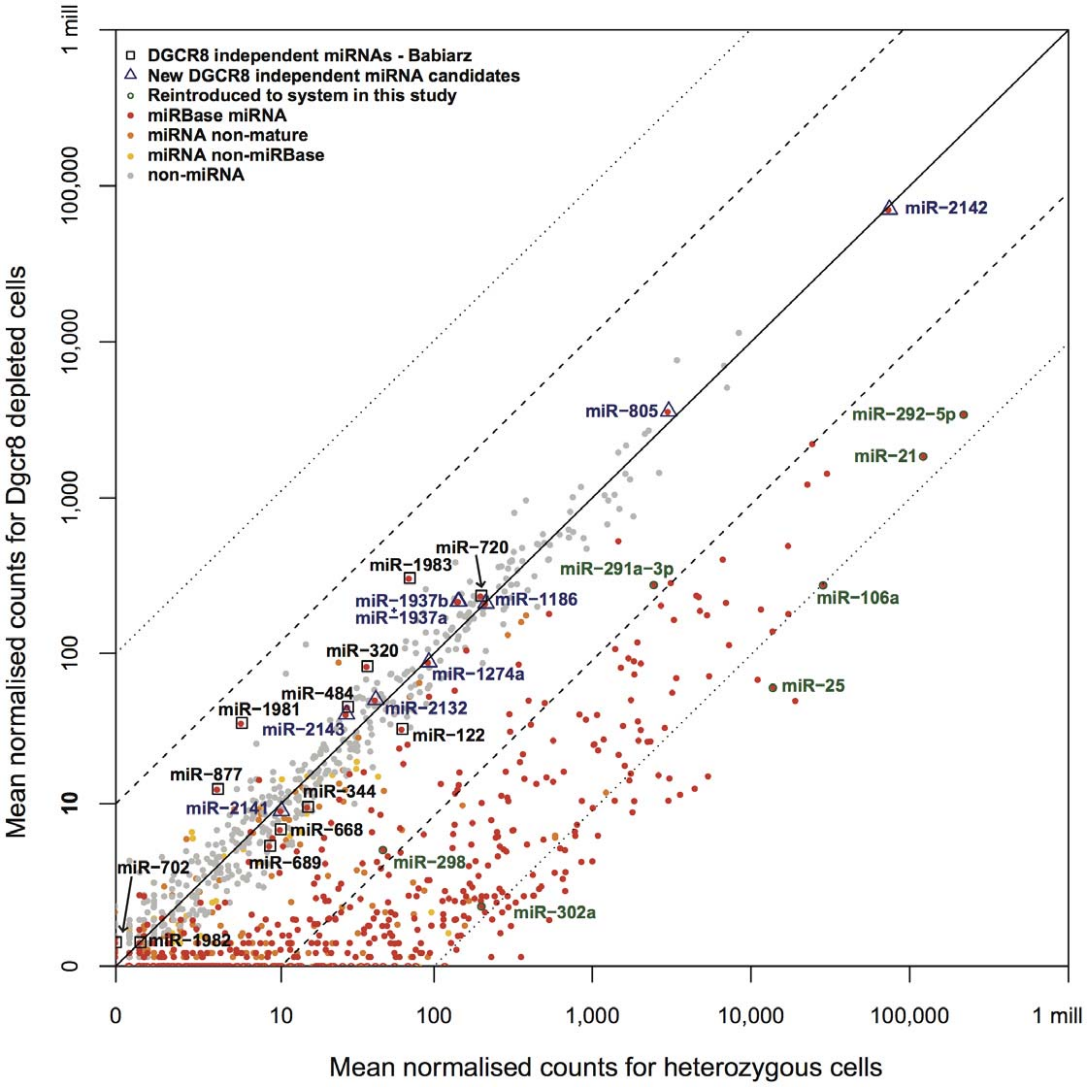
Mean normalised mapped read counts for non-coding RNAs in the heterozygous and homozygous mutant cells. Highlighted in green are those miRNAs reintroduced by transfection as part of this study. Those miRNAs highlighted in black are the miRNAs proposed by Babiarz *et al.* as *Dgcr8*-independent miRNAs [25]. Highlighted in blue are those miRBase miRNAs with an average of more than 10 reads in the heterozygote cell lines but for which the expression is down-regulated less than 1.5 fold between the heterozygous and homozygous mutant cell lines. These potentially represent further *Dgcr8*-independent miRNAs. The dashed and dotted lines represent 10 fold and 100 fold expression changes between cell lines respectively.

Following normalization it was observed that there is a substantial reduction in the expression of miRNAs in the *Dgcr8^gt1/tm1^* and *Dgcr8^gt2/tm1^* cell lines (*Dgcr8*-depleted cells) (Figure 1 and Figure S4B). This confirms the functional effect of the two *Dgcr8* gene trap cassettes inserted into the *Dgcr8* locus and the disruption of DGCR8 function. There was a slight reduction in the normalised levels of miRNAs in the heterozygous cell lines when compared to the wild-type ES cells, although this is clearly not of a magnitude that approaches the reduction seen in the *Dgcr8*-deficient cells (Figure S4B). This reduction could be caused by a haploinsufficiency of *Dgcr8* leading to the reduced processing of primary miRNA transcripts in the heterozygotes.

### Effect of miRNA depletion on mRNA expression

To better understand the molecular consequences of miRNA depletion, mRNA expression was assayed for each of the cell lines. A comparison between WT and the individual heterozygous cell lines identified only 10 and 62 differentially expressed transcripts (in the two replicates respectively). This demonstrates that there is no broad effect on mRNA expression of heterozygous *Dgcr8* depletion. In contrast, independent comparisons of the two homozygous mutant cell lines to their corresponding heterozygous control line identifies 2220 and 3101 transcripts with significantly altered expression, with approximately 73% of the smaller set of transcripts also found within the larger set. Thus, homozygous depletion of *Dgcr8* results in a large number of significant expression changes at the mRNA level and those changes are highly consistent between replicates.

**Figure 2:**
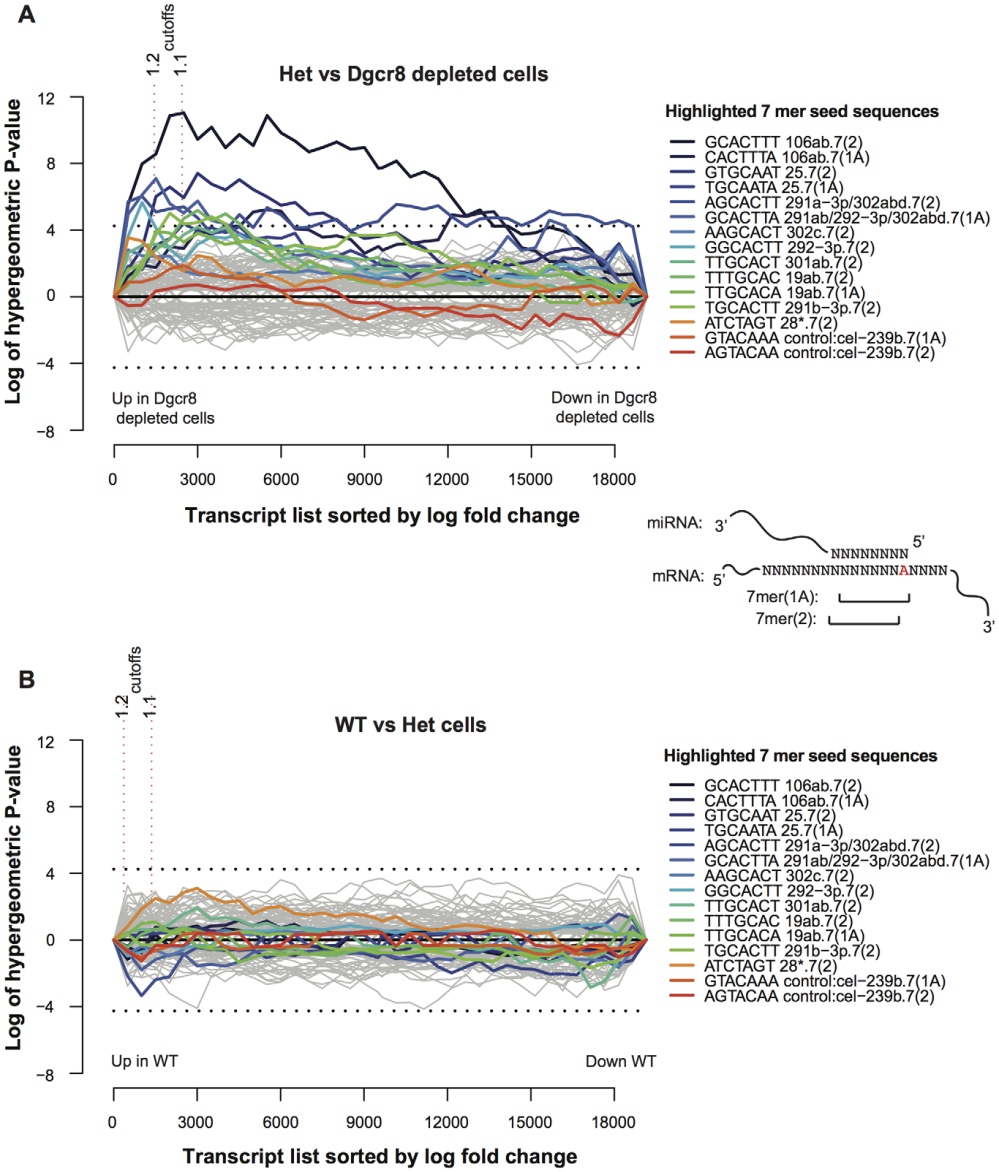
Sylamer plots of miRNA seed enrichment amongst genes whose expression changes following *Dgcr8* depletion. **A**) Heterozygote vs. *Dgcr8*-depleted cells. Independent homozygous mutant and heterozygous cell lines are considered replicates. Array probes and associated transcripts were sorted according to log fold change in *Dgcr8^gt1/tm1^* and *Dgcr8* ^gt2/tm1^ cells compared to *Dgcr8^tm1,gt1/+^*and *Dgcr8* ^tm1,gt2/+^ cell lines (see Materials and Methods). These lists were used for Sylamer analysis. The x-axis represents ordered transcripts possessing a 3′ UTR sequence. Up-regulated transcripts in the homozygous mutant cells are to the left and down-regulated to the right. Each grey line represents enrichment or depletion of individual miRNA 7mer seeds in 3′ UTRs to the left of a data point relative to the right of the data point. Positive scores on the y-axis indicate probes to the left of this point are associated with enrichment of seed sequences. Negative scores indicate depletion. Seed sequences of particular interest are highlighted in colour. Each highlighted seed sequence is annotated with numbers corresponding to associated miRBase miRNAs (eg. 106), letters representing corresponding miRNA family members (eg. ab) and the seed sequence type (eg. 7(2)). Enrichment and depletion is tested at progressive intervals of 500 transcripts. Red vertical dotted lines indicate a fold change cut off. Horizontal black dotted lines represent a Bonferroni-corrected P-value threshold of 0.01. **B**) An equivalent analysis comparing the expression of WT cells to Heterozygote cells. For this analysis, *Dgcr8^gt1/tm1^* and *Dgcr8* ^gt2/tm1^cells were considered as replicates and compared to their WT cell line.

We used the Sylamer algorithm for statistical analysis of miRNA seed matches within mRNA expression gene lists [26]. The expectation is that miRNA targets will be up-regulated in *Dgcr8*-depleted cells. Sylamer analysis of the list of transcripts ordered according to mRNA expression change (log fold change) upon *Dgcr8* depletion (see Materials and Methods) identifies a clear enrichment of highly expressed ES cell miRNA seed sequences amongst those genes most significantly up-regulated in the *Dgcr8^gt1/tm1^* and *Dgcr8^gt2/tm1^* cell lines (Figure 2A). This demonstrates the functional consequences of *Dgcr8* depletion on ES cell expressed miRNAs and de-repression of direct miRNA targets. By contrast a comparison of the heterozygote expression profile to that of the WT cell lines revealed no significant enrichment for miRNA seed sequences within any regions of a gene list ordered by fold-change (Figure 2B).

In order to ensure that changes in expression upon *Dgcr8* depletion are not simply caused by differentiation, WT, heterozygous and *Dgcr8*-depleted cells were tested by Western Blot for Oct4 expression (an ES cell marker [27]) (Figure S6). As expected all cell lines clearly expressed this marker. This is consistent with previous studies that have noted that the disruption of the miRNA-processing pathway in mouse ES cells does not lead to their differentiation [18]–[20].

As heterozygotes did not appear to be significantly effected at the mRNA level following the disruption of a single allele, yet had been treated in the same fashion as the *Dgcr8*-depleted cells throughout this study, *Dgcr8*-depleted cells were compared to the heterozygote cells lines for the remainder of the analyses.

### *Dgcr8*-independent miRNAs

It has been previously noted by Babiarz *et al.* [25] that there are a number of miRNAs whose expression does not appear to be affected by the depletion of functional DGCR8 and these have therefore been proposed as *Dgcr8*-independent miRNAs. These miRNAs behave in a similar fashion in this study, and are relatively unaffected by the homozygous mutation of *Dgcr8* (marked by square boxes in Figure 1). In addition to the miRNAs previously described there is also evidence to support the *Dgcr8*-independent processing of a number of additional miRNAs (Figure 1). Of these, miR-2142 appears to be the most highly expressed. Indeed, closer inspection of this miRNA has revealed that it overlaps with a 5S ribosomal sequence and likely does not represent a true miRNA sequence. As such it raises the possibility that a proportion of other *Dgcr8*-independent miRNAs reported in miRBase may represent mis-annotation of other non-coding RNA species. Subsequently, 8 out of 9 of the small RNAs newly predicted here as potential *Dgcr8*-independent miRNAs (including miR-2142) have been removed from miRBase (v18 Table 1). Our results are consistent with previous studies in the field while identifying an additional candidate for *Dgcr8* independence. Furthermore, they highlight a requirement for robust miRNA annotation with the advent of next-generation sequencing.

**Table 1:** Candidate *Dgcr8*-independent miRNAs.

Dgcr8 independent miRNA candidates	Enzymatic Dependency observed	Fold Change	Still in miRBase v18	Reason cited in miRBase for removal*
mmu-miR-1186	**This study**	***0.985***	Yes	
mmu-miR-2142	**This study**	***0.950***	**No**	**“fragment of 5S rRNA” This study**
mmu-miR-805	**This study**	***1.186***	**No**	“overlaps a Mt tRNA”
mmu-miR-1274a	**This study**	***0.948***	**No**	“fragment of a Lys tRNA (Schopman et al.)^1^”
mmu-miR-1937a	**This study**	***1.500***	**No**	“fragment of tRNA”
mmu-miR-1937b	**This study**	***1.516***	**No**	“fragment of tRNA”
mmu-miR-2132	**This study**	***1.174***	**No**	“fragment of rRNA (Chiang et al.)^2^”
mmu-miR-2143	**This study**	***1.447***	**No**	“fragment of 28S rRNA”
mmu-miR-2141	**This study**	***0.897***	**No**	“fragment of rRNA (Chiang et al.)^2^”
mmu-miR-720	Babiarz et al.	***1.178***	Yes	
mmu-miR-1983	Babiarz et al.	***4.324***	Yes	
mmu-miR-320	Babiarz et al.	***2.172***	Yes	
mmu-miR-122	Babiarz et al.	0.519	Yes	
mmu-miR-484	Babiarz et al.	***1.581***	Yes	
mmu-miR-1981	Babiarz et al.	***5.821***	Yes	
mmu-miR-344	Babiarz et al.	0.645	Yes	
mmu-miR-877	Babiarz et al.	***3.105***	Yes	
mmu-miR-668	Babiarz et al.	***0.688***	Yes	
mmu-miR-702	Babiarz et al.	***1.421***	Yes	
mmu-miR-1982	Babiarz et al.	***0.995***	Yes	
mmu-miR-689	Babiarz et al.	0.631	**No**	“fragment of rRNA (Chiang et al.)^2^”
mmu-miR-699	Babiarz et al.	NA	**No**	“fragment of RNase MRP RNA”

Presented are the candidate *Dgcr8*-independent miRNAs alongside those identified as *Dgcr8*-independent by Babiarz *et al.* [25]. Included is the fold change of each miRNA seen in this study between heterozygous and *Dgcr8*-depleted cell lines (bold italics if they meet our fold change threshold) and the status of each miRNA in miRBase version 18.

* Reasons for removal from miRBase (http://www.mirbase.org/) as given in miRBase, except for miR-2142, which was identified for removal by this study (^1^ [42], ^2^ [41]).

### Identification of miRNA targets through reintroduction of miRNAs

A system depleted of the vast majority of miRNAs provides an opportunity for the identification of miRNA targets in a clean background [28]. The targets of individual miRNAs will no longer be saturated by endogenously expressed miRNAs allowing a more thorough investigation of target interactions by miRNA transfection assays. Furthermore, such experiments will not encounter problems associated with functional redundancy of related miRNAs that may impede miRNA knockout and knockdown assays. To identify miRNA targets, miRNA mimics were transfected into *Dgcr8^gt1/tm1^* cells and the mRNA expression profile of the cells was subsequently assayed in relation to cells transfected with a control duplex (see Materials and Methods).

**Figure 3:**
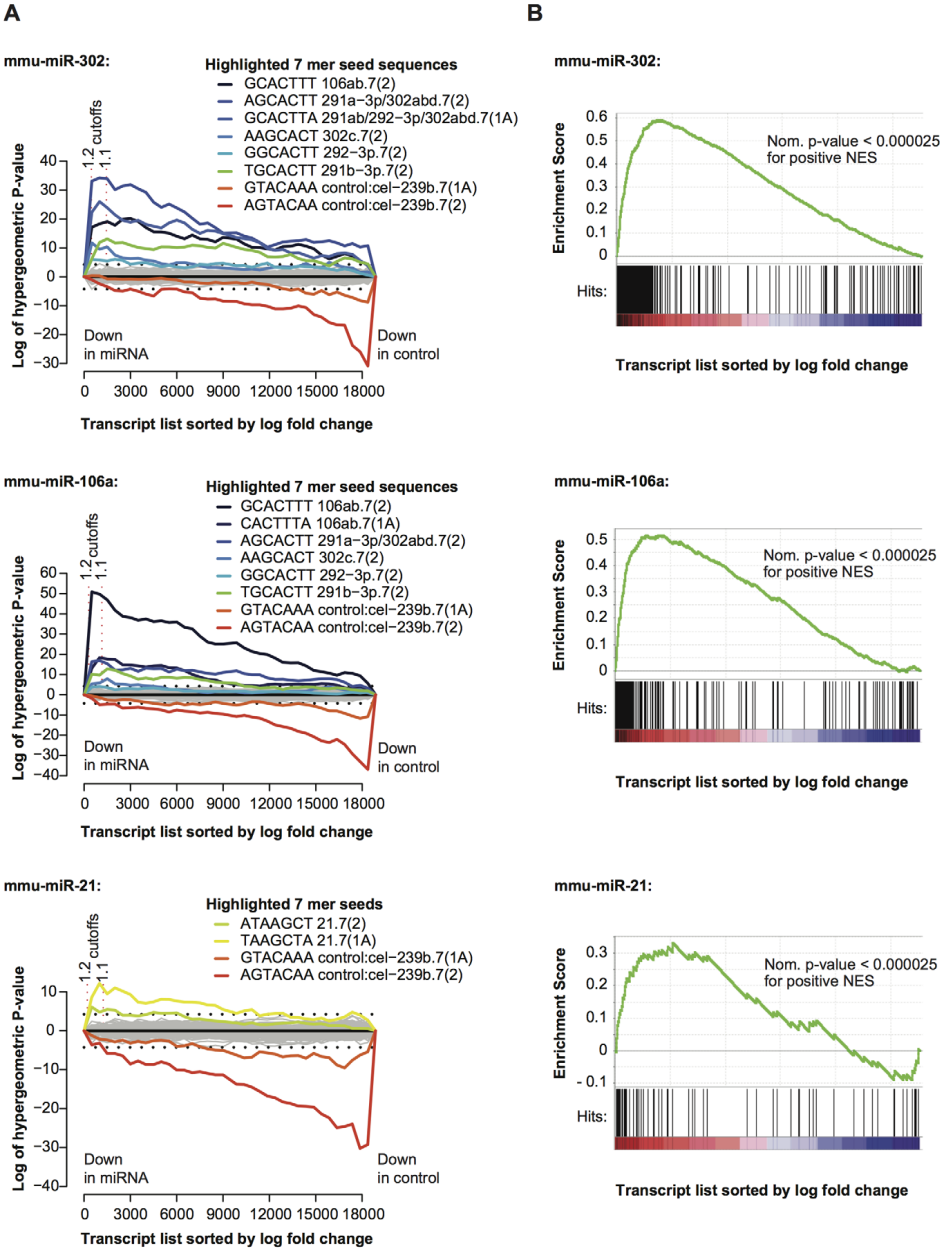
The use of global expression profiles to determine miRNA-dependent transcriptional effects. **A**) Sylamer analysis of expression profiles following the transfection of miRNA mimics into *Dgcr8* ^gt1/tm1^ cells. Array probes and associated transcripts were ordered according to their log fold change in those cells transfected with a miRNA mimic (miR-302a, miR-106a, and miR-21) when compared to those transfected with a control duplex (cel-miR-239b). These lists were then subjected to the Sylamer analysis (See Figure 2 for a description of Sylamer plots). Transcripts relatively down regulated in the cells transfected with the test miRNAs are to the left, while those relatively down-regulated in the controls are to the right. **B**) GSEA enrichment plots [29] depicting the enrichment of the transcripts within the miRNA target lists for miR-302a, miR-106a and miR-21 within regions of a list of transcripts ordered according to expression following the depletion of *Dgcr8*. The relative expression of transcripts in heterozygous cell lines compared *Dgcr8*-depleted cell lines is plotted on the x-axis, ordered according to log fold change, with those genes up-regulated in homozygous mutant cell lines to the left. Black lines on the horizontal axis represent the positions of the miRNA targets within the ordered transcript lists. The green line represents the “running” enrichment score at each position progressing through the gene list. If the maximum deviation of the “running” enrichment score from 0 is positive this implies an enrichment of miRNA targets amongst those genes up-regulated in the *Dgcr8*-depleted cells. Conversely if the maximum deviation is negative the targets are relatively enriched amongst down-regulated genes.

A set of miRNAs was transfected including miR-25, miR-291a-3p, miR-292-5p, miR-106a, miR-21, miR-302a and miR-298. These miRNAs were selected based on a variety of criteria including expression in mouse ES cells, depletion upon the disruption of *Dgcr8*, shared or contrasting seed sequences or a propensity to enhance the induction of induced pluripotent stem (iPS) cells (Table 2). In all cases, except miR-292-5p, there is an enrichment of corresponding seed sequences within the UTRs of the genes down-regulated by the miRNA transfection (P-value: <0.01) (Figure 3A and Figure S7A). In the case of miR-292-5p the enrichment for the corresponding seed sequences failed to reach this level of significance. The significant enrichment seen in the majority of cases demonstrates that the transfection of miRNA mimics was successful and the regulatory effect of the mimics is readily apparent at the mRNA level. Significantly down-regulated genes were selected and these were incorporated into the target list for the miRNA if the 3′ UTR of the corresponding transcript contains at least one 7mer(1A) or 7mer(2) seed sequence for the relevant miRNA (Table S1).

**Table 2:** Features of miRNAs used in this study.

miRNA	8mer(1A)	Expressed in ES cell (% of miRBase mature mapped reads)	Evidence for role in iPS cell generation for miRNA or family	Seed sequences enriched upon *Dgcr8* depletion
**mmu-miR-298**	CCTCTGCA	0.002	YES† ^,^ 1	NO
**mmu-miR-291a-3p**	**AGCACTTA**	0.547	YES† ^,^ 2	YES
**mmu-miR-302a**	**AGCACTTA**	0.060	YES† ^,^ 1 ^,^ 2	YES
**mmu-miR-292-5p**	GTTTGAGA	21.094	YES† ^,^ 1	NO
**mmu-miR-21**	ATAAGCTA	18.087	YES§ ^,^ 3	NO
**mmu-miR-25**	GTGCAATA	1.186	YES† ^,^ 1	YES
**mmu-miR-106a**	*GCACTTTA*	5.992	YES† ^,^ 2	YES

Seed sequences highlighted in bold represent seeds common to multiple miRNAs. The seed highlighted in italics possesses a 7mer(3) seed equivalent to the 7mer(2) seed of the bold seed sequences.

† iPS cell promoting miRNAs:

1 D. Lu *et al.* [49],

2 R. Sridharan *et al.* [62].

§ miRNAs that inhibit iPS cell generation:

3 C.-S. Yang *et al.* [63].

### Target list quality

In order to judge the effectiveness of these experiments in generating significant miRNA target lists, calculations were performed to estimate the signal to noise ratio of each and an estimate was made of the number of targets in the target list above that which may have been expected by chance (Table S2). The signal to noise ratio varied form 11.8∶1 for the miR-25 target list to 2.2∶1 for the miR-292-5p target list (Table S2). In addition, the number of targets generated above expected, varied between 242 target transcripts for miR-302a to 20 target transcripts for miR-291a-3p (Table S2). Taken together with the Sylamer analysis described above, these results indicate that the target lists provided by this study include a large number of true miRNA targets.

The transfection of individual miRNAs into the *Dgcr8^gt1/tm1^* cells should represent the reversal of the effect of the depletion of *Dgcr8*, at a molecular level. To confirm this, the expression profile of the *Dgcr8*-depleted cell lines when compared to the heterozygous controls was interrogated with each miRNA target list in turn, using Gene Set Enrichment Analysis (GSEA) [29] (Figure 3B and Figure S7B). In all cases except miR-292-5p and miR-298 there is clear enrichment of the miRNA targets of each miRNA in the genes up-regulated when miRNAs are depleted, confirming that the gene lists represent the effects of a reversal of *Dgcr8* depletion. Hence, the regulation of transcripts within these gene lists is likely to be highly relevant in an ES cell context.

There are several factors that may account for the differences in both the length of the target lists generated for each miRNA and the observed enrichments. The target lists of miR-302a and miR-106a are both the longest and most enriched amongst the genes up-regulated upon broad miRNA depletion in the ES cell system (by normalised enrichment score (NES)). By contrast miR-292-5p and miR-298 have weaker seed sequence enrichments amongst significantly down-regulated genes following transfection and their proposed targets are not enriched among the genes up-regulated by *Dgcr8* depletion. It is possible that the networks regulated by these miRNAs differ in complexity or they regulate a different number of *in vivo* targets. These enrichments may be more easily identifiable for miRNAs with large numbers of targets, a more significant effect on the down-regulation of those targets or which cause a broad depletion of an entire network, therefore having a more pronounced cumulative effect. On the other hand the seed enrichment for those miRNAs with fewer targets, whose role is to maintain homeostatic regulation of those targets under specific circumstances, or whose target networks may be corrected by feedback loops following perturbation may be harder to detect in this way. In the case of miR-298, it is not highly expressed in mouse ES cells so we were not expecting to identify a large number of *in vivo* targets that would be up-regulated upon miRNA depletion.

### Overlap between ES cell miRNA targets

It might be expected that ES cell expressed miRNAs regulate similar processes and may have considerable overlap between their target sets, leading to a robust layer of post-transcriptional regulation. It is expected that miRNAs with shared seed sequences will also share a considerable number of their targets due to the extent to which the recognition of miRNA target sites depends on the degree of complementarity between the target transcript and the miRNA seed region [30]. In order to identify coordinated regulation and potential functional redundancy amongst the miRNAs tested we compared the target lists of each miRNA (Figure S8). The functional redundancy expected between the targets of miR-291a-3p and miR-302a, which share a seed sequence, is clearly evident. These two miRNAs share 86% of the miR-291a-3p target transcripts. This large overlap is contrasted with the much lower overlap (0–11%) seen between these two miRNAs and miR-298, miR-21, miR-25 and miR-292-5p (Figure S8). This implies that although ES cell expressed miRNAs may regulate similar pathways in the cell they must do so by targeting a broadly differing set of transcripts.

Intriguingly, miR-106a also shares a shifted seed sequence (7mer(3)) with both miR-291a-3p and miR-302a (7mer(2)), which may also suggest that they maintain functionally redundant roles through interaction with the same target sites. However, the transcripts bearing only the 7mer(3) seed sequence of miR-106a do not appear to be significantly affected by the transfection of miR-106a mimics into the *Dgcr8^gt1/tm1^* cells (Figure S9), so it is not expected that the miRNAs will affect identical target sequences. It is noteworthy, however, that miR-106a does share 41 to 52% of its targets with miR-302a (Figure S10A). In addition the majority of the targets shared by both miR-106a and miR-302a do contain an 8mer sequence that will be complementary to the seed regions of both miR-106a and miR-302a (AGCACTTT), rather than simply distinct target sites for each miRNA (Figure S10B). Thus in these specific cases redundancy may be maintained.

### Ascribing functional roles to the miRNAs

**Figure 4:**
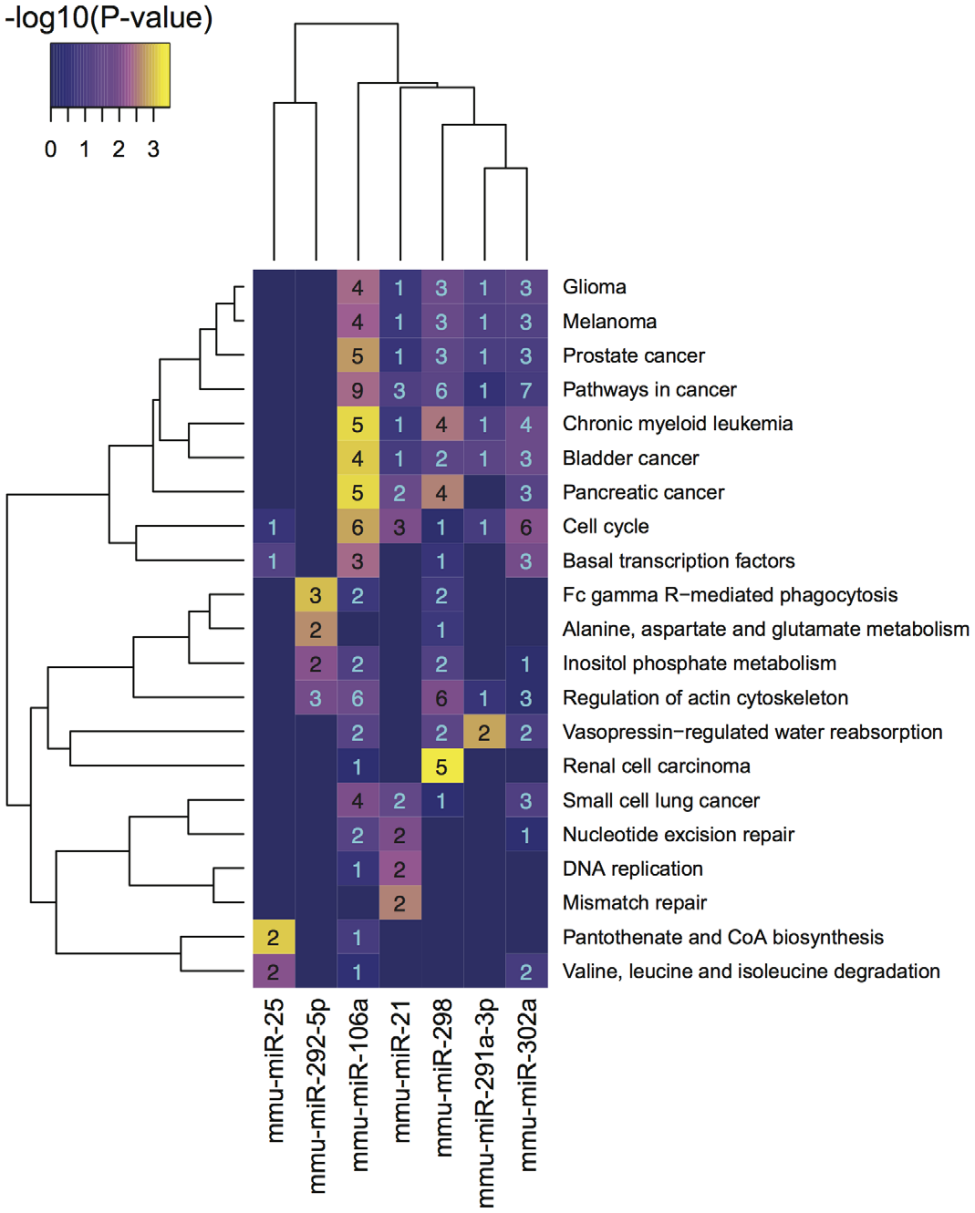
Kegg Pathways significantly enriched in the targets of at least one miRNA. Represented are KEGG pathway terms found to be enriched in at least a single miRNA target list with a P-value less than 0.01. The purple to yellow colour gradient represents the relative significance of the enrichment in each case (−log_10_(P-value)). The numbers represent the number of target genes whose annotation contributes to the pathway enrichment. For a full description of the significantly enriched pathways see Table S3.

In order to better understand the function that each of these miRNAs play in mouse ES cells, enrichment analysis of annotation to Kyoto Encyclopedia of Genes and Genomes (KEGG) pathways [31] was performed upon the miRNA target lists (Figure 4). This analysis confirmed previously identified roles for several of the miRNAs. For example the targets of miR-106a are significantly enriched in pathways relating to cancer. This miRNA is a member of a family that has been strongly associated with cancer [32]. In addition miR-298, miR-302a, miR-21 and miR-291a-3p appear to target genes in these pathways to varying degrees. Of these, miR-302a, miR-21 and miR-291a-3p, or their human homologues, have all been associated with cancer [33], [34]. Mouse ES cells bearing mutations in the miRNA processing pathway had an extended G1/S phase phenotype that was rescued by transfection of members of miRNA families that include miR-106a, miR-302a and miR-291a-3p [22]. Our results contain 11 unique targets within the KEGG *cell cycle* network. Indeed, the targets of both miR-106a and miR-302a are significantly enriched within this category. Although the targets of miR-291a-3p are not enriched in this pathway, this is not surprising given the relatively small size of the miR-291a-3p target list. Our results therefore support a fundamental role for miRNAs in regulating the ES cell cycle.

Our miR-106a target list includes *Ccnd1, Cdkn1a* and *E2f1*, all of which have been shown to targeted by the miR-17 family of miRNAs which includes miR-106a [35]–[37]. *Cdkn1a*, a key regulator of the G1/S phase transition, also appears in our miR-302a and miR-291a-3p target lists. These miRNAs share a seed sequence and as such this is consistent with the results of Wang *et al.* [22]. Furthermore, Cyclin D1, a confirmed target of miR-302a in human ES cells [38] is within the miR-302a target list presented here. The miR-25 target list contains a single gene within the *cell cycle* pathway, *Cdkn1c*, a confirmed miR-25 target [35]. Thus our target lists are recapitulating a number of previously observed miRNA-target relationships, leading us to believe that other targets within the list are worthy of further consideration.

It is intriguing to note that multiple miRNAs appear to converge on a number of genes in the *cell cycle* pathway. Considering the targets for those miRNAs either highly expressed in WT ES cells or for whom there is a clear seed sequence enrichment amongst those genes up-regulated following *Dgcr8* depletion (Table 2), *Cdkn1a* (miR-291a-3p, miR-302a and miR-106a) and *E2f2* (miR-106a, miR-302a and miR-21) both appear to be potentially targeted by three miRNAs, while *Skp2* (miR-21 and miR-302a) and Cyclin D1 (miR-106a and miR-302a) may be targeted by two each. Further to this, miR-302a appears to target multiple members of the anaphase promoting complex. This implies a degree of cross-regulation and redundancy not only between miRNAs with shared seed sequences (miR-291a-3p and miR-302a) but also both amongst miRNAs with no shared seed identity and through the regulation of multiple components of a functional complex.

While miR-302a and miR-106a appear to make the greatest contribution to regulation of the cell cycle, the targets of miR-21 are also significantly enriched in this pathway despite possessing a completely different seed sequence. The connection of miR-21 to cell cycle regulation is perhaps expected as this miRNA is known to have altered expression in a number of cancers. It is clear from these interactions and the additional associations demonstrated in this study, that a complex network of miRNAs regulate the progression of the ES cell cycle.

**Figure 5:**
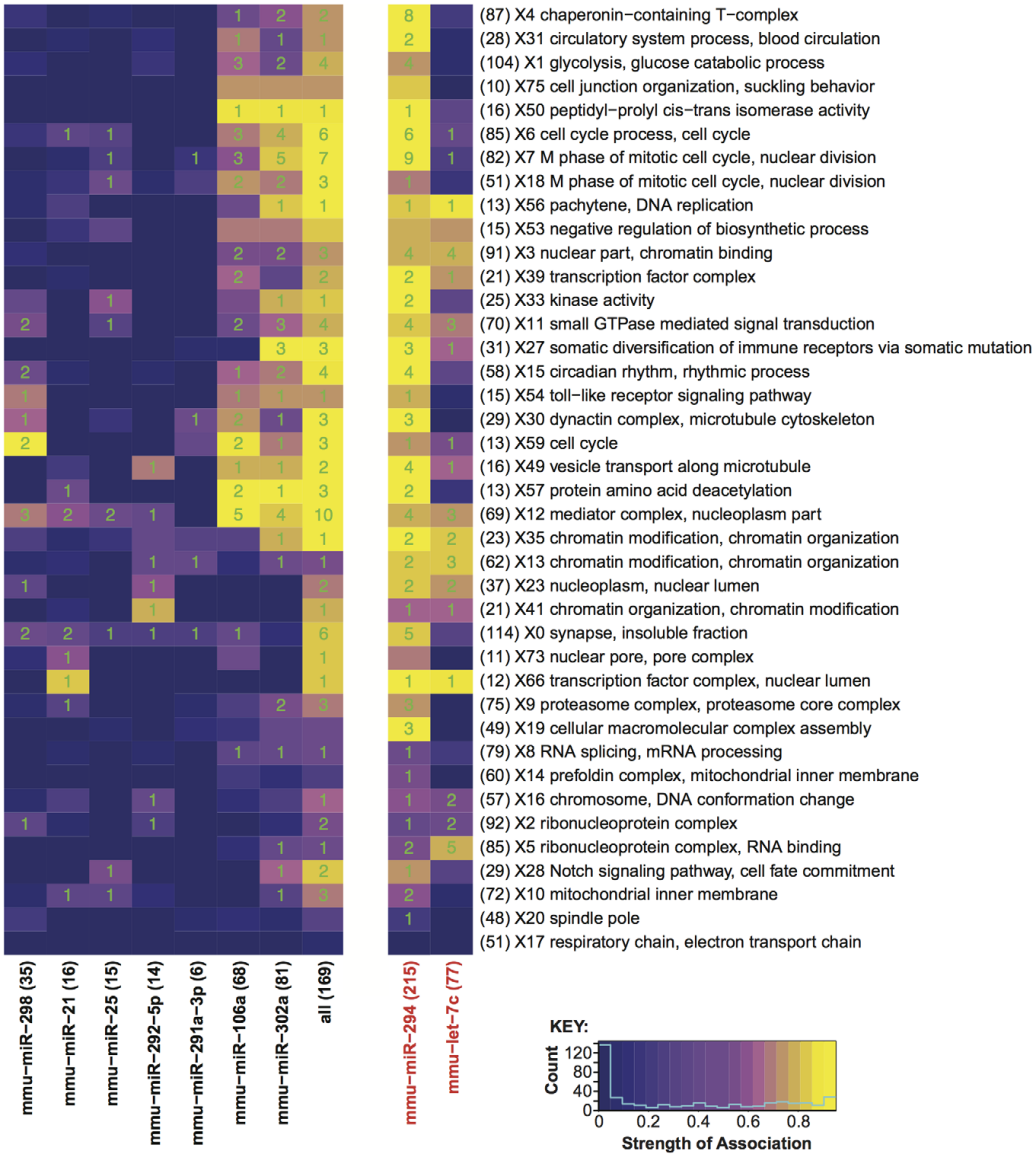
Influence of the targets of individual miRNAs on the mouse molecular interaction network. Rows represent individual protein clusters derived from the Pathway Commons interaction network. Each cluster was functionally annotated using Gene Ontology enrichment analysis (Right). The most enriched GO Terms are displayed. Clusters are numbered in descending size order (X#), the number of genes in each cluster are recorded in parentheses. Each column within the heat map represents an individual miRNA target set. Those miRNAs in black represent gene lists that were derived as part of this study, those in red represent gene lists derived from Melton *et al.* [40] (see Materials and Methods). The ‘all’ list is a combined set of targets represented in any of the lists derived as part of this study. For each target list the number of genes represented in the interaction network are recorded in parentheses. The relative influence of the targets of each miRNA upon each cluster is ascribed according to the number of nodes adjacent to miRNA-targeted nodes, which reside within a given cluster (see Materials and Methods). The purple to yellow gradient reflects increasing miRNA influence. The green numbers represent the number of genes targeted by the specified miRNA within the respective protein cluster.

The predicted miRNA targets were mapped to a molecular interaction network to extend the analysis beyond annotated KEGG pathways and facilitate interpretation of larger networks. This network was subsequently clustered (see Materials and Methods) and functions were assigned to each cluster through Gene Ontology (GO) term enrichment analysis (Figure 5). The miRNA targets predicted in this study influence some regions of the network more heavily. Unsurprisingly, many of these clusters were related to the cell cycle, which is consistent with both the KEGG analysis presented above and previous works. Perhaps more intriguing was the large number of targets concentrated within cluster 12. This cluster contains 69 proteins, 10 of which appear to be targeted by one or more of the miRNAs presented here (Figure S11 and S12). This cluster is comprised mainly of components of the mediator complex but also associated complexes (e.g. PolII, ATAC and general transcription factors). The mediator complex is thought to act as a site of signal integration, coordinating the interaction of the pre-initiation complex (containing PolII) with transcriptional activators and repressors [39]. It is via the apparent regulation of components of the pre-initiation complex that miR-106a targets are enriched in the *basal transcription factors* KEGG Pathway (Figure 4).

In order to further explore the role miRNAs may play in the regulation of the mediator complex and confirm that our findings can be replicated in other studies we selected those genes with a 3′ UTR seed match identified as significantly down-regulated by the miR-294 and let-7a in the work of Melton *et al.* [40]. We examined the enrichment of genes within these lists amongst the clusters of the interaction network (Figure 5). Again, these gene lists appear to be enriched within cluster 12. Intriguingly, despite their opposing roles in the regulation of self-renewal in ES cells, both of these miRNAs would appear to converge upon this sub-network (Figure S11) [40].

Our results suggest that miRNA mediated regulation of this central hub of transcriptional regulation may play a significant role in both maintenance of ES cells and formation of iPS cells.

## Discussion

In this study we have presented a comprehensive experimental approach to miRNA target detection in ES cells. The generation of a *Dgcr8*-deficient cell line provides an excellent system for the reintroduction of miRNAs for target identification against a “clean” background. As a consequence of developing this system, we have also demonstrated that miR-1186 is potentially processed in a *Dgcr8*-independent manner.

Recently miRBase has begun to make its criteria for miRNA annotation more stringent [13]. This should allow more accurate annotation of miRNAs based on RNA-seq data. We have demonstrated that systems such as that presented here can be used to interrogate miRBase to shortlist miRNA annotations worthy of further scrutiny. Indeed, similar efforts have begun, testing the maturation of overexpressed miRNA hairpins in the context of dominant-negative alleles of Drosha or Dicer [41]. As we have noted, a number of small RNAs that appeared to be processed in a *Dgcr8*-independent manner have recently been removed from miRBase. These small RNAs all appear to overlap either ribosomal or tRNA genes. While it is clear that the correct classification of small RNA fragments can be a complicated process [42], small RNAs of disparate origin may possess miRNA-like function [43]. As such our work clearly highlights a current and multifarious issue facing the field of miRNA biology that will require considerable additional work to resolve.

As ES cell expressed miRNAs are sufficient for the induction of pluripotency [44], [45] and clearly influence the expression of a broad range of cellular pathways (Figure 5), it is remarkable that miRNAs are not essential for the maintenance of the ES cell self-renewal and the expression of marker genes [18]–[20]. These observations imply a set of complex interactions between miRNAs and the other regulators of pluripotency. Indeed, such networks between miRNAs and core ES cell transcription factors have been established [46]. It also appears highly likely that the specific context of a broad set of miRNA-target interactions will profoundly affect miRNA function. Further reconciling these observations requires significant work. The removal of the expression of all miRNAs from the mouse ES cell system suggests that miRNAs are critical for transitioning to a differentiated state and unnecessary in the maintenance of self perpetuating ES cells, but it will most likely be through the disruption and overexpression of the individual, highly redundant, miRNA gene families that the intricacies of miRNA modulated pluripotency can be unpicked.

Through the analysis of the potential targets identified in this study for a number of miRNAs, it is clear that miRNAs interact within complex networks often displaying overlapping roles and considerable functional redundancy both at the gene and nucleotide levels. In the light of this, attempts to understand miRNA function through the perturbation of single genes may appear daunting. It is through the modeling and understanding of large-scale miRNA-target interactions that the complete picture of miRNA mediated regulation can be realised. This requirement for a global understanding of miRNA function is further confounded by the relative strength of individual miRNA-target interactions and the effect that this can have on the function of a miRNA in context [47].

miRNAs do not only regulate cellular expression at the level of their primary targets but also through the regulation of influential secondary targets. It has long been known that miRNAs regulate transcription factors and influence transcriptional regulation via these intermediaries [48]. In addition, we have recently demonstrated that miR-25 directly targets two significant ubiquitin ligases and may influence the core ES transcriptional network as a consequence [49]. This adds a further abstraction to miRNA-mediated regulation. This role of miRNAs in regulating ubiquitin ligases has also been recently demonstrated by others [50]–[52]. The results presented here also imply that miRNAs may influence global cellular expression at a far more basal level. Indeed, miRNA-mediated alterations in the expression of components of the mediator complex and other core transcriptional complexes could have wide ranging implications on the functions of many diverse cellular pathways. The exact mechanisms by which the mediator complex influences gene expression are currently the topic of intense research [39], [53]–[55], and confirming and disentangling the role of miRNAs at this basal level clearly requires substantial further work, but it is hoped that the targets highlighted here may provide a guide for such investigations. Indeed, the disruption of the mediator complex in ES cells has been demonstrated to lead to transcriptional and morphological changes consistent with the loss of the ES cell state and differentiation [55]. Hence, understanding the role that miRNAs may play at this level will be essential to our understanding of both maintenance of self renewal and the formation of iPS cells.

## Materials and Methods

### Generation of mutant cell lines

Two independently derived cell lines were acquired from BayGenomics (XG058 and XH157), each with a gene trap in a single allele of *Dgcr8*, within the intron between exons 9 and 10 (ENSMUST00000115633). To validate the clones, nested RT-PCR reactions were performed with primers in the gene trap cassette sequence and exon sequences upstream of the insertion site (Gene specific: 5′-TACGGATCTGGAACTGCAAG, 5′-CTCAAGGTCCGCCCTGTTTA. Gene trap cassette: 5′-ATTCAGGCTGCGCAACTGTTGGG, 5′-AGTATCGGCCTCAGGAAGATCG). PCR products were subsequently purified and sequence confirmed. Each of the cell lines was subcloned and reconfirmed by RT-PCR.

*NotI* digested BAC clone bMQ-62C21 [56] was used as the template for the amplification of a 6 kb homology region by long PCR (Expand 20 kb PLUS PCR System (Roche)), using primers that contained an *AscI* restriction site (5′-AATTGGCGCGCCCCTGGAGTAGGCATGTTGATTTCAC, 5′-AATTGGCGCGCCATGCTGAGACAAGACTGGAAACCAC). The cloning strategy for the *Dgcr8* insertion-type targeting construct is shown in Figure S1. The *AscI* fragment contains exons 4–8 of *Dgcr8* and was cloned into pR3R4_AsiSI, replacing the chloramphenicol resistance gene and ccdB cassette. The fragment was then transferred to the pL3/L4_(+)_GT1T2hygroP2EGFP gene trap plasmid through an *in vitro* L/R clonase reaction (Gateway L/R clonase II (Invitrogen)). Prior to electroporation, the plasmid was linearised in the homology region at a unique *HindIII* site in intron 6 of *Dgcr8* (ENSMUST00000115633). Hygromycin resistant clones were picked and expanded. Nested RT-PCR between *Dgcr8* specific primers that bind to exons upstream of both the gene trap insertion sites and homologous region and primers that are common to both gene trap cassettes were used to detect the trapped and targeted alleles (Gene specific: 5′-GCTGCAGGAGTAAGGACAGG, 5′-GTGGATGAAGAGGCCTTGAA. Gene trap cassette: 5′-TTCTTTGGTTTTCGGGACC, 5′-GTTTTCGGGACCTGGGAC). Clones that express both the BayGenomics gene trap and the targeted gene trap alleles were considered to be homozygous mutant lines. Products representative of each of the expected amplicon sizes were confirmed by sequencing.

### Cell culture

Cell lines were maintained feeder-free in ES cell medium (GMEM+10% serum+LIF) at 37°C, 7% CO_2_ in gelatinised tissue culture treated plates and flasks and electroporated as described [57]. Medium was supplemented with selective agents dependent upon cell genotype. *Dgcr8^+/+^* (E14) [57], cells were cultured in non-selective media; *Dgcr8^gt1/+^* and *Dgcr8^gt2/+^* cells were maintained in media supplemented with 150 µg (active)/ml G418 (Geneticin - Gibco). Following the electroporation of the gene-targeting construct, cells were selected at 120 µg/ml Hygromycin B (Calbiochem) and maintained at 100 µg/ml Hygromycin B. To assay for β-galactosidase activity, cells were washed with PBS, fixed in 0.2% gluteraldehyde and stained with 1 mg/ml Xgal as described [57]. Cells were imaged at a magnification of 10× with relief contrast. For standard expression array profiling, unless otherwise stated, cells were cultured for 4 days in non-selective media and lysed with Trizol while sub-confluent.

### RNA blotting

Cells were maintained for 4 days in non-selective media prior to lysis. RNA was purified by Trizol and RNeasy according to the manufacturers protocol. RNA was quantitated on an Agilent Technologies 2100 Bioanalyser using a Eukaryotic Total RNA Nano Chip. Polyadenylated RNA was purified with the PolyATract mRNA Isolation System III (Promega) and quantitated as above to ensure removal of rRNA. Sample concentrations were equalized, and separated by denaturing agarose gel electrophoresis in the presence of SybrGreen (Invitrogen) and a 0.5–10 kb RNA ladder (Invitrogen). Separated RNA was transferred overnight by capillary blot to Hybond XL membrane (GE Healthcare) and UV cross-linked (UVP).

RNA probes were prepared by amplification from *Dgcr8^+/+^* Trizol-purified-RNA derived cDNA followed by subcloning and reamplification. Probes were amplified with the KOD Hot-Start PCR kit (Novagen) using primers GCTGGGCTGTTGTCTCCATA and CATCTTGGGTTTCTTCCGAGT (3′) or CGACGACCCATTCAACTTCT and TCGAGCACTGCATACTCCAC (5′) and purified by Qiagen Qiaquick Gel Extraction. The probe fragments were A-tailed (NEB Buffer, dATP (Amersham), AmpliTaq (Perkin Elmer), ∼250 ng DNA), and cloned using pGEM-T-Easy (Promega) with the Roche Rapid Ligation Kit and MACH1 cells (Invitrogen). Transformant colonies were confirmed by colony PCR with flanking plasmid primers GTAAAACGACGGCCAGT and GGAAACAGCTATGACCATG. Plasmids were prepared using a Qiaprep Spin Miniprep Kit (Qiagen) and inserts were confirmed by sequencing. The probes were finally amplified by KOD Hot-Start PCR and the probe specific primers and purified by Qiaquick Gel Extraction (Qiagen).

Amplified probes were radiolabeled using the Random Labeling kit (Invitrogen). Hybridisations were performed overnight in PerfectHybTMPlus buffer (Sigma) at 55°C. The membrane was washed to 0.1× SSC with 0.1% SDS at 55°C and imaged by phosphoimager (GE Healthcare) for 2.5 hours.

### Western blot

Cells were maintained for 4 days in non-selective media prior to protein purification and lysed while subconfluent. The cells were lysed in protein lysis buffer (50 mM Tris HCl pH7.5, 0.5 M NaCl, 1% IGEPAL CA-630, 1% Sodium Deoxycholate, 0.1% SDS, 2 mM EDTA, Complete Protease Inhibitors (Roche)) by repeated passage through a 21 G hypodermic needle. Samples were quantified with Bradford Reagent. 50 µg of each reduced sample was size separated on a 4–12% Bis-Tris Gel (Invitrogen). The proteins were transferred to a Hybond-ECL filter. Oct4 and α-tubulin were detected using primary antibodies (Santa Cruz sc8628, Abcam ab7291, respectively) and peroxidase conjugated secondary antibodies (Sigma-Aldrich A4174 and A6782) were used to visualise each protein sequentially with Western Lightning reagents.

### miRNA mimic transfection protocol

*Dgcr8^gt1/tm1^* cells were cultured for 4 days in non-selective media and plated to gelatinised 6 well plates at 96×10^4^ cells per well. Cells in each well were transfected after 3 hours. 240 pmoles of miRNA mimic were added to 240 µl OptiMEM I (Gibco). 7.2 µl of Lipofectamine 2000 (Invitrogen) was added to further 240 µl of OptiMEM and incubated for 5 minutes at room temperature. Both solutions were mixed gently and then combined. This mixture was incubated for a further 25 minutes at room temperature. The media on the cells was replaced with 2.4 ml of fresh, non-selective media. The miRNA-lipid complexes in the OptiMEM mixture were then transferred to this media and the wells were mixed gently. 5 hours later, the media was aspirated from the cells and replaced with a further 7.2 ml of non-selective media. 10 hours after the initiation of transfection the cells were lysed for RNA with 1 ml of Trizol (Invitrogen). Duplicate miRNA transfections were performed and expression profiles were compared to those of control mimic transfections prepared in parallel.

To determine the optimal time post-transfection at which to lyse cells to identify the gene set most enriched for primary miRNA effects, transfected cells were lysed in a time-series post-transfection and their mRNA expression was profiled at each time point. Initially, miR-291a-3p and miR-25 were transfected into the *Dgcr8^gt1/tm1^* cells. Both of these miRNAs are highly expressed in the wild type mouse ES cells, are down-regulated in the *Dgcr8^gt1/tm1^* and *Dgcr8^gt2/tm1^* cell lines and their seeds are enriched in the 3′ UTRs of transcripts up-regulated upon *Dgcr8* depletion. The Sylamer algorithm was used to analyse gene lists ordered according to differential expression between the miRNA transfected cells and those transfected with a control duplex. This identified 10 hours as the time point at which the seed sequences for each miRNA were most significantly enriched amongst the down-regulated genes.

### miRNA mimics

miRIDIAN Negative Control #2 (Dharmacon CN-002000-01-05)

miRIDIAN mmu-miR-291-3p mimic (Dharmacon C-310470-01-0005)

miRIDIAN mmu-miR-25 mimic (Dharmacon C-310564-01-0005)

miRIDIAN mmu-miR-302 mimic (Dharmacon C-310483-05-0005)

miRIDIAN mmu-miR-292-5p mimic (Dharmacon C-310471-03-0005)

miRIDIAN mmu-miR-106a mimic (Dharmacon C-310488-07-0005)

miRIDIAN mmu-miR-21 mimic (Dharmacon C-310515-05-0005)

miRIDIAN mmu-miR-298 mimic (Dharmacon C-310479-07-0005)

### Expression arrays

Trizol purified RNA was cleaned up with an RNeasy MiniElute Cleanup Kit (Qiagen). 500 ng of RNA was amplified and labeled with the Illumina Total Prep RNA Amplification Kit (Ambion). Microarrays were processed by the Sanger Institute Microarray Facility. Briefly, 1500 ng of biotinylated cRNA was hybridised to Illumina expression BeadChips (Mouse-6 v1.1 for mmu-miR-291-3p and mmu-miR-25 mimics and cell line expression profiles, and Mouse-6 v2 for mmu-miR-302, mmu-miR-292-5p, mmu-miR-106a, mmu-miR-21 and mmu-miR-298 mimics. Independent Negative Controls were analysed on both arrays). Bead chips were processed following the manufacturer's instructions and analysed using BeadStudio software v.3.1.8 or GenomeStudio version 1.1.1 (Illumina). All array data has been submitted to ArrayExpress (E-MTAB-418 and E-MTAB-968).

### Computational analysis of expression arrays

Analysis of the expression array data was conducted in R/Bioconductor (http://www.bioconductor.org/) with additional packages (*lumi*, *limma, org.Mm.eg.db, gplots, RColorBrewer, Vennerable, KEGG.db, Biobase, R2HTML*). Samples derived from each array version were combined into 2 independent sets, variance-stabilising transformation (VST) transformed and quantile normalised [58] using the *lumi* package of Bioconductor. Probes from both the v1.1 and v2 formats were mapped to Ensembl gene annotation v56 (preferentially annotating each probe to a VEGA transcript and then Ensembl transcripts with the longest annotated 3′ UTR). Additional annotation for these transcripts, including Gene ID, Entrez ID, Gene symbol and Description were also derived from Ensembl. When no Ensembl transcript could be found for a probe, a RefSeq transcript was assigned if available from Illumina chip annotation along with additional annotation (MouseWG-6_V1_1_R4 and MouseWG-6_V2_0_R2). Probes with no transcript annotation were removed form the set used for transcriptional analysis. Where multiple probes mapped to the same target transcript, the probe with the highest inter-quartile range across arrays was retained. The remaining probes were considered for differential expression between samples. Samples ascribed to different array versions and arrays relating to miRNA transfection and broad cell line profiling experiments were analysed independently at this stage.

Differential gene-expression analysis was carried out on these data by performing an empirical bayes t-test with Benjamini Hochberg multiple-testing correction via the *limma* package of Bioconductor. When identifying significantly altered expression between wild type, heterozygote and *Dgcr8*-depleted samples a fold-change of 1.2 and a p-value (adjusted for FDR) cutoff of 0.1 were used where required.

Transcripts were considered as possible miRNA targets if they were significantly down-regulated at least 1.2× (P-value below) in the presence of a transfected miRNA mimic relative to the expression in cells transfected with a control duplex. Since the v2 arrays appear to be more sensitive, a p-value cutoff of 0.05 was used for these miRNA transfection experiments, compared to 0.10 for the v1.1 arrays. Annotation information was assigned to the significant probes according to Ensembl v56.

For the purposes of this study 7mer(1A), 7mer(2) and 7mer(3) and 8mer(1A) seed sequences were defined as follows:

7mer(1A): A 6 nucleotide sequence complementary to positions 2 to 7 of the miRNA followed by an adenosine.

7mer(2): A 7 nucleotide sequence complementary to positions 2 to 8 of the miRNA

8mer(1A): A combination of the two seed sequences above.

7mer(3): A 7 nucleotide sequence complementary to positions 3 to 9 of the miRNA

Sylamer was used to count the number of corresponding miRNA seed sequences within the unmasked 3′ UTRs of annotated target transcripts. Transcripts without one or more corresponding 7mer(2) or 7mer(1A) seed sequence were removed from the target lists.

### Sylamer analysis

Sylamer was used to verify miRNA-directed changes of gene expression. The expectation is that targets of many miRNAs will be up-regulated upon *Dgcr8* depletion. In the comparisons between each miRNA transfection and the corresponding control, the targets of the miRNA should be relatively down-regulated by the miRNA transfection. Heterozygote and homozygous mutant samples derived from either the *Dgcr8^gt1/+^* and *Dgcr8^gt2/+^* lineages were considered as replicates for the comparison of expression between *Dgcr8*-depleted cells and heterozygotes and heterozygote and WT cells.

Briefly, Sylamer tests for miRNA effects by searching sorted lists of genes for enrichment or depletion of all possible words complementary to the seed regions of miRNAs. The method uses the hypergeometric statistic, comparing the counts of each word in all the 3′ UTR sequences, before a cutoff in a sorted gene list, to the counts in the remaining genes after that cutoff. Gene lists were produced by sorting the transcripts with annotated 3′ UTR sequence (see Computational analysis of Expression Arrays), according to the observed differential expression between different samples. Sylamer parameters used were -k 7 (words of length 7), -grow 500 (analyse the gene list in growing steps of 500 sequences), -m 4 (use Markov correction for nucleotide biases using observed frequencies of words of length 4) and -words word_file (only test words present in ‘word_file’). The words considered were all the 7 nt words complementary to positions 1–8 of annotated mouse miRNAs for which at least 10 reads were sequenced in any of our Illumina GA experiments, always using an adenine as the base matching the first position of the miRNA [59]. In total, 568 distinct words were considered. All 3′ UTR sequences were pre-processed to remove low-complexity and duplicated sequences as described previously [26].

### Estimates of target list quality

As a measure of the specificity of this target identification method a signal to background ratio was determined for each miRNA target list. The proportion of transcripts that were down-regulated significantly upon the introduction of the miRNA, which possess a miRNA seed in their 3′ UTR (7mer(2) or 7mer(1A)) was divided by the proportion of transcripts whose expression was altered less than 1.1 fold in the same experiment (control set) and which possess the same seeds in their UTRs. Similarly the sensitivity of each target list was calculated as the number of significantly down-regulated transcripts that possess a seed sequence for the specific miRNA beyond the number of transcripts that would be expected given the proportion of transcripts with seeds in the control set.

### KEGG pathway analysis

Probes used in the initial expression analyses were mapped to Entrez IDs (based on Ensembl v56 annotation or Illumina annotation files (see above)) and used as a Gene Universe. For this analysis probes without Entrez Gene ID annotation were removed in addition to duplicate IDs. Furthermore, Entrez IDs without any associated KEGG annotation (according to the Bioconductor package *org.Mm.eg.db*) were removed from the corresponding analyses. After filtering, the genes belonging to each miRNA target list were compared to the rest of the genes from the corresponding array using the *GOstats* package to test for enriched KEGG pathways. A p-value significance cutoff of 0.01 was used.

### GSEA analysis of miRNA target transcripts in relation to changes following the depletion of miRNAs from an ES cells system

Gene Set Enrichment Analysis (GSEA) was performed using the “GSEAPreranked” method [29]. For a subset of entities within a ranked ‘universe’ the method estimates the significance of the enrichment of the subset within any region of the ranked distribution. The transcripts in the ‘universe’ consisted of all of the transcripts queried by the initial analysis for differential expression, ordered according to log fold expression change in the comparison of transcript expression between *Dgcr8^tm1,gt1/+^* and *Dgcr8^tm1,gt2/+^* cell lines and *Dgcr8^gt1/tm1^* and *Dgcr8^gt2/tm1^* cells (see above). The gene subsets tested for enrichment consisted of the miRNA target lists derived from the transfection of the miRNA mimics (see above). Where target lists were derived from experiments performed on an array version that differed from that used in the comparison of expression in heterozygous cell lines to the *Dgcr8*-depleted cells, only transcripts whose expression was interrogated by both array versions were retained for this analysis. The number of permutations used in the GSEA analysis was 40000, which allows for the estimation of a minimum p-value detection of 2.5E-5.

### Interaction network analysis

The molecular interaction network was retrieved from Pathway Commons (Feb 2011) [60]. Nodes annotated with a human or mouse taxonomic ID were retained. Nodes without an Entrez ID were removed. Subsequently NCBI annotation was used to convert the nodes with human Entrez IDs to their mouse counterparts. Where multiple potential Entrez IDs were present, those found within a mouse miRNA target list were selected in preference. In other cases the ‘first’ Entrez ID was chosen. Subsequently the Pathway Commons network was converted to a series of mouse Entrez ID delineated edges.

For each miRNA, targets without an annotated Entrez ID were removed from the lists along with duplicate Entrez IDs. The miR-294 and let-7a target sets were derived from Melton *et al.* Supplementary Table 1 [40]. Genes down-regulated following the transfection of either miR-294 or let-7a and with one or more corresponding seed sequence in their 3′ UTR were selected for further analysis. Genes with no symbol were removed. Where a gene was annotated with multiple symbols the first was considered. Duplicate symbols were collapsed. Symbols were converted to Entrez IDs using the *org.Mm.eg.db* library in R/Bioconductor. In the case of multiple Entrez IDs the first was selected. All NAs and duplicate IDs were removed from the gene sets.

The initial interaction network consisted of 4808 nodes with 55894 edges, corresponding to an average node degree of 23.3. In this network, there were many nodes of very high degree, namely two nodes of degree > = 400, 4 nodes of degree > = 300, 28 nodes of degree > = 200, and 218 nodes of degree > = 100. Such ‘hub’ nodes obscure cluster structure, as they tend to pull together many nodes into coarse clusters.

This network was reduced using a k-nearest neighbour approach, with k chosen as 100. With this approach, an edge E with weight w between two nodes a and b is kept only if w is in the top k edge weights for 1) the edges emanating from a and 2) the edges emanating from b. The input network could not be submitted to k-NN (k-nearest neighbour) reduction straight away, as it is a simple network with all edge weights equal to 1. The chosen approach was to add to each edge weight a number proportional to the number of triangles in which the edge participates. This preprocessing step, promoting edges in accordance with the number of secondary connections, allowed the application of k-NN reduction. Following this reduction with k = 100, the resulting network has 46788 edges, corresponding to an average node degree of 19.5. In the resulting network the highest node degree is now 100.

The reduced network (which we shall call the k100 network) was submitted to MCL clustering. Two clusterings were considered, at different levels of granularity, with the first a superclustering of the second. The first, coarse clustering was obtained by using inflation 1.3, the second was obtained by subclustering the k100 network restricted to the first clustering, with inflation set to 2.0. The clusters were annotated by running a GO-enrichment script written in R, using the customary hypergeometric test. All GO categories (MF, CC, BP) were considered. The second clustering was deemed more informative, as large clusters split into smaller units with more specific annotation. For example, the largest supercluster with 433 nodes and annotations ‘ribonucleoprotein complex (8.7e-71), ‘RNA splicing’ (1.1e-47) and ‘RNA processing’ (2.5e-47) split into a cluster with 92 nodes (‘ribonucleoprotein complex’, 6.1e-24), a cluster with 85 nodes (‘ribonucleoprotein complex’, 2.0e-28), a cluster with 85 nodes (‘cell cycle process’, 3.4e-11), a cluster with 82 elements (‘M phase of mitotic cell cycle’, 1.2e-26), a cluster with 79 elements (‘RNA splicing’, 4.9e-67) and several smaller clusters. When considering the fifty largest clusters in the second clustering, the median of the best P-value associated with each of the clusters (referring to a GO-term enriched for such a cluster) is 6.811652e-10. For each cluster, the two best scoring GO-terms were used to annotate the cluster in the heatmap (Figure 5). For pragmatic reasons, if the display length of these two terms exceeded 50 characters, only the best term was used.

The heatmap shows for each list of target genes (corresponding to a list of nodes in the network) and for each cluster the support for that list in that cluster. This number (the support) is the sum of the support of each individual node (from the list) for that cluster, normalised by cluster size. The support of a node is the sum of edge weights (in the k100 network) for its edges that connect it to (other nodes in) the cluster, divided by the total sum of weights of all its outgoing edges.

It is thus possible for a cluster to give support to a node that is not part of the cluster, simply by virtue of the node having neighbours in that clusters. Additionally, the heatmap shows numbers in the cells. Such a number indicates, for the cluster and target list specific to that cell, the number of genes shared between the cluster and the list.

### miRNA expression profiling using Illumina/Solexa high-throughput sequencing

Cells were maintained for 4 days in non-selective media prior to lysis. RNA was purified with Trizol (Invitrogen). Large RNA was removed from the small RNA fraction using the RNeasy Mini Kit (Qiagen), and small RNAs were collected by isopropanol precipitation and quantitated on an Agilent Technologies 2100. Sequencing libraries were prepared by an adapted version of Illumina “Preparing Samples for Analysis of Small RNA” protocol version 1 (2007). Initially, the 3′ adaptor was added to heat denatured small RNA fraction and ligated with RNA ligase in the presence of 20% DMSO and RNase Out (Invitrogen). The RNA between 35–65 bp was size separated on a Novex 15% TBE-urea gel, eluted and ethanol precipitated in the presence of GlycoBlue (Ambion). Heat denatured RNA was ligated to the 5′ Adapter as before. RNA of 60–100 bp in size was purified by 10% TBE-urea gel electrophoresis and recovered as above. The RNA was reverse transcribed and the cDNA was amplified by PCR using the smRNA primer 2 and RT primer (Illumina) with Phusion Taq (NEB). Heat denatured cDNA was purified by 10% SequaGel PAGE gel electrophoresis and recovered in 0.3 M NaCl followed by ethanol precipitation and quantified by Agilent Technologies 2100 Bioanalyzer. The Illumina libraries were Solexa sequenced by the Sanger Institute Core Sequencing Facility (36-cycle Single-ended run, Illumina GA instrument). Sequencing data has been submitted to ArrayExpress and ENA (E-MTAB-975).

### Computational analysis of the high-throughput sequencing data

The *FASTQ* files from each sequencing lane were processed by R/Bioconductor using the *ShortRead* and *Biostrings* packages. After verifying that the sequencing quality was acceptable for all libraries, the 3′ adapter sequence was trimmed from all the reads with the *trimLRPatterns* function. The distribution of read lengths confirmed the expected enrichment of miRNAs (a peak of ∼22 nucleotides) for the wild type and heterozygous samples, while this was essentially absent in the *Dgcr8*-depleted samples. Reads were then trimmed if they contained runs of ambiguous nucleotides (three Ns in a window of five bases) of if they contained any number of Ns within 2 bases from the end of the read. A low-complexity filter was implemented to remove all reads that consisted mostly of runs of identical nucleotides, di-nucleotides or tri-nucleotides. The remaining sequences of lengths between 16–30 were kept for further analysis, collapsing them into unique sequences and keeping track of the observed depth of sequencing.

A dataset of all mouse non-coding RNA sequences was obtained by extracting all the hairpin sequences from miRBase v14 and all RNA genes and predictions from Ensembl v56. The distinct sequence reads were mapped against this dataset using SSAHA2 [61]. Minimum requirements for valid alignments consisted of 90% identity, 16 bases of length and coverage of 75% of the read. The reads had to be aligned starting from either position 1 or 2, but no restriction was put on the 3′ end due to non-templated nucleotide addition. No gaps were allowed in the alignments and reads mapping to the reverse-complement strand were ignored. Using Ensembl annotation, reads were classified according to the type of ncRNA that they mapped to into tRNA, snRNA, snoRNA, rRNA, Mt tRNA, Mt rRNA, misc RNA and miRNA. Those that mapped to more than one class were separated into a “mixed mapping” group. The miRNA reads were cleaned further by separating those mapping outside the mature miRNA region (miRNA non mature) and those mapping to miRNAs not present in miRBase (miRNA non miRBase).

The normalisation strategy consisted of using the total depth of reads mapping to ncRNAs (excluding the miRNA and mixed categories) and scaling all the libraries to have an equivalent depth to the WT sample (Table S4).

## Supporting Information

Figure S1 **Schematic illustration of the gene targeting strategy used in this study.** **A**) Map of vectors introduced in this study. The pR3R4AsiSI plasmid is a Gateway shuttle vector for cloning genomic DNA fragments. Positive and negative selection cassettes, CmR and ccdB, respectively are flanked by *AscI* restriction sites and attR3 and attR4 Gateway sites. The pL3L4_(+)_GT1T2hygP2EGFP plasmid contains a gene trapping cassette and attL3 and attL4 Gateway cloning sites to allow transfer of cloned genomic DNA fragments. The gene trap cassette is composed of the En2 splice acceptor, hygromycin resistance gene (HygroR), Enhanced Green Florescent protein gene (EGFP) and the SV40 polyadenylation site (SV40 polyA). The T2 and P2 sites cause ribosome skipping and are included for optimal expression of the resistance marker and fluorescent reporter [64]. **B**) Cloning of the homology region into the pL3L4_(+)_GT1T2hygP2EGFP vector. See Materials and Methods for a detailed description of the cloning strategy. The resulting targeting vector is named pGT1T2hygP2EGFP_Dgcr8. **C**) Schematic of the *Dgcr8*-targeted trapped and gene trapped alleles. The pGT1T2hygP2EGFP_Dgcr8 plasmid is an insertion-type gene-targeting vector containing exons 4 to 8 of *Dgcr8*. The vector is linearised within the homology region at a unique *HindIII* site prior to electroporation. The resulting targeted events cause a duplication of the homology region, placing the hygromycin-EGFP cassette downstream of exon 8. The BayGenomics gene trap cassette contains a β-geo reporter cassette, conferring G418 resistance and β-galactosidase activity, inserted downstream of exon 9. Insertion of the targeted cassette into the Bay Genomics gene trap allele will silence the β-geo reporter (Figure S2).
(TIFF)

Figure S2 **Xgal staining of cell lines to determine β-geo (β-galactosidase) activity associated with the initial gene trap.** Xgal-staining confirmed that in the selected heterozygous cell lines (*Dgcr8^tm1,gt1/+^* and *Dgcr8^tm1,gt2/+^*), the insertion of the second gene trap had disrupted the expression of the fusion transcript containing the original downstream construct and silenced the β-galactosidase activity of the fusion protein produced. This staining demonstrated that both gene traps are inserted within the same allele of the target gene. In contrast the homozygous mutant cell lines retained positive x-gal staining confirming that the gene traps must be within separate alleles of the gene. The inserted pane shows the nuclear localisation of the β-galactosidase activity of the β-geo fusion protein.
(TIFF)

Figure S3 **RNA blot of** ***Dgcr8*** **derived transcripts assessing the expression of wild type and fusion transcripts.** RNA derived from the two heterozygous cell lines were separated (*Dgcr8^tm1,gt1/+^* and *Dgcr8^tm1,gt2/+^*) alongside the 2 homozygous mutants (*Dgcr8^gt1/tm1^* and *Dgcr8^gt2/tm1^*) and the wild type cell line (*Dgcr8* ^+/+^). Additionally, RNA samples from the parental gene trap cell lines were also blotted (*Dgcr8^gt1/+^* and *Dgcr8^gt2/+^*). The blot was hybridised sequentially with radiolabelled probes that either anneal to the 5′ (top) or 3′ (bottom) ends of the *Dgcr8* transcripts. The expected transcript sizes are shown to the right of the blot. Size estimates for the gene-trapped transcripts are based on ENSMUST00000115633 and include the gene trap cassettes up to the polyadenylation sites. The nature of the smaller wild type transcript is unclear although the second small wild type transcript has previously been observed in mouse [65].
(TIFF)

Figure S4 **Read counts for each class of ncRNA in each cell line.** **A**) Raw small RNA mapped read counts. **B**) Equivalent read counts after scaling to the non-miRNA non-coding RNA population in the WT sample.
(TIFF)

Figure S5 **Total read counts for each RNA species compared between cell lines pre-normalisation.**
(TIFF)

Figure S6 **Western blot comparing the expression of Oct4 in** ***Dgcr8*** **-depleted, heterozygous and wild type cell lines.** The same blot was treated with antibodies for both Oct4 and α-tubulin (loading control).
(TIFF)

Figure S7 **Global expression profiles to determine miRNA-dependent transcriptional effects.** **A:** Sylamer plots comparing the expression profiles of *Dgcr8* ^gt1/tm1^ cells transfected with a miRNA mimic (miR-25, miR-291a-3p, miR-292-5p or miR-298) and a cel-miR-239b control miRNA. For a full description see Figure 3. **B:** GSEA enrichment plots [29] judging the enrichment of the transcripts within the miRNA target lists for miR-25, miR-291a-3p, miR-292-5p or miR-298 within regions of a list of transcripts ordered according to log fold change following the depletion of *Dgcr8* in homozygous mutant cell lines. For a full description see Figure 3.
(TIFF)

Figure S8 **Overlap in target transcripts between each of the miRNAs.** The percentage overlap represents the proportion of the transcripts within the smallest target set which overlap the larger set in each pairwise comparison between miRNA target sets. Bonferroni corrected hypergeometric P-values were calculated for each overlap. The universe consisted of those transcripts interrogated in the differential expression analyses. The number of potential targets identified for each miRNA is recorded beneath the miRNA name. In cases where targets were derived from alternative microarray versions, only transcripts interrogated by both platforms were considered in the comparison.
(TIFF)

Figure S9 **Assessing the potential for functional redundancy between miR-106a and miR-302a through a shifted 7mer(3) seed.** A cumulative graph is presented of the relative effect of each miRNA seed sequence (7mer (1A), 7mer (2) and 7mer (3)) on transcripts which contain at least one seed from the relevant category and for which the seed is not part of a longer seed matching site (achieved through the exclusion of transcripts containing adjacent 7mers) (Red, green and blue lines) when compared to a 1/10 sampling of all transcripts represented on the array (Black), following the transfection of miR-302a or miR-106a miRNA mimics. P-values displayed were calculated using a Wilcoxon or T-test to determine if the relative distribution of the seed bearing transcripts, according to log fold change (logFC), differ significantly from the bulk of the other transcripts following miRNA transfection. The x-axis represents the relative logFC following miRNA transfection when compared to the cel-miR-239b. The y-axis is the cumulative percentage of each target set or transcripts represented on the array.
(TIFF)

Figure S10 **Investigation of the seed sequences found in miR-106a and miR-302a targets.** **A**) A Venn diagram of transcripts within the target lists of both miR-106a and miR-302a. Shown are the numbers of transcripts found exclusively within the miR-302a target list and the miR-106a target list in addition to those shared by both. **B**) The class of seed sequence found in the 3′ UTRs of target transcripts. The nature of the miR-106a and miR-302a seed sequences found within the 3′ UTRs of the transcripts found exclusively in the miR-106a target list, the miR-302a target list or those transcripts within both target lists. The extended 8mer refers to the target sequence AGCACTTT, which is complementary to the accepted seed sequences of both miR-302a and miR-106a. The miR-302a and miR-106a target sites exclusively refer to those target sites that do not overlap these extended sites and are therefore not expected to be targeted by both miRNAs.
(TIFF)

Figure S11 **miRNAs found to target transcripts of proteins found within cluster 12 of the interaction network.** Hexagons represent proteins and grey lines represent known interactions. Stars mark those genes in the sub-network targeted by miRNAs either in this study or in the work of Melton *et al.* [40] (see Materials and Methods).
(TIFF)

Figure S12 **Relative disruption of miRNA targets within mediator associated cluster.** Log fold change of transcripts associated with the miRNA targets from within cluster 12 (and Med7, a mediator protein missing from the cluster) upon the addition of each miRNA relative to the control miRNA (Left) and the log fold change upon the depletion of all miRNAs (Right). Stars represent those transcripts selected as potential targets of the transfected miRNA.
(TIFF)

Table S1 **Transcripts identified as probable miRNA targets.** Transcripts identified as the probable targets of each miRNA over-expressed within this study. Each table includes the number of relevant seed sequences within the transcript's 3′ UTR, the log fold change and associated p-value of each transcript following transfection of the miRNA when compared to a cel-miR-239b control miRNA (see Materials and Methods) and the corresponding target prediction scores provided by TargetScan (v5.0) [4], MirTarget2 (v3.0) [17] and microT (v3.0) [16]. For a more complete description of miR-25 targets please see D. Lu *et al.* [49].
(XLSX)

Table S2 **Estimates of the signal to noise ratio of each of the target lists produced as part of this study.**
(XLSX)

Table S3 **KEGG Pathway analysis results.** Significant KEGG pathway terms over-represented amongst the targets of the miRNAs with a significance cutoff of 0.01 in each case.
(XLSX)

Table S4 **Sequences mapping to RNA class sets.** A summary of the number of sequences mapping to each RNA class set. Columns in bold italics represent those RNA classes used to normalise the samples.
(XLSX)
