# Supplementary figures and images for: Large-Scale Identification of MicroRNA Targets in Murine Dgcr8-Deficient Embryonic Stem Cell Lines
Source: PLoS One. 2012 Aug 17;7(8):e41762. doi: 10.1371/journal.pone.0041762 (PMC3422281; doi:10.1371/journal.pone.0041762)

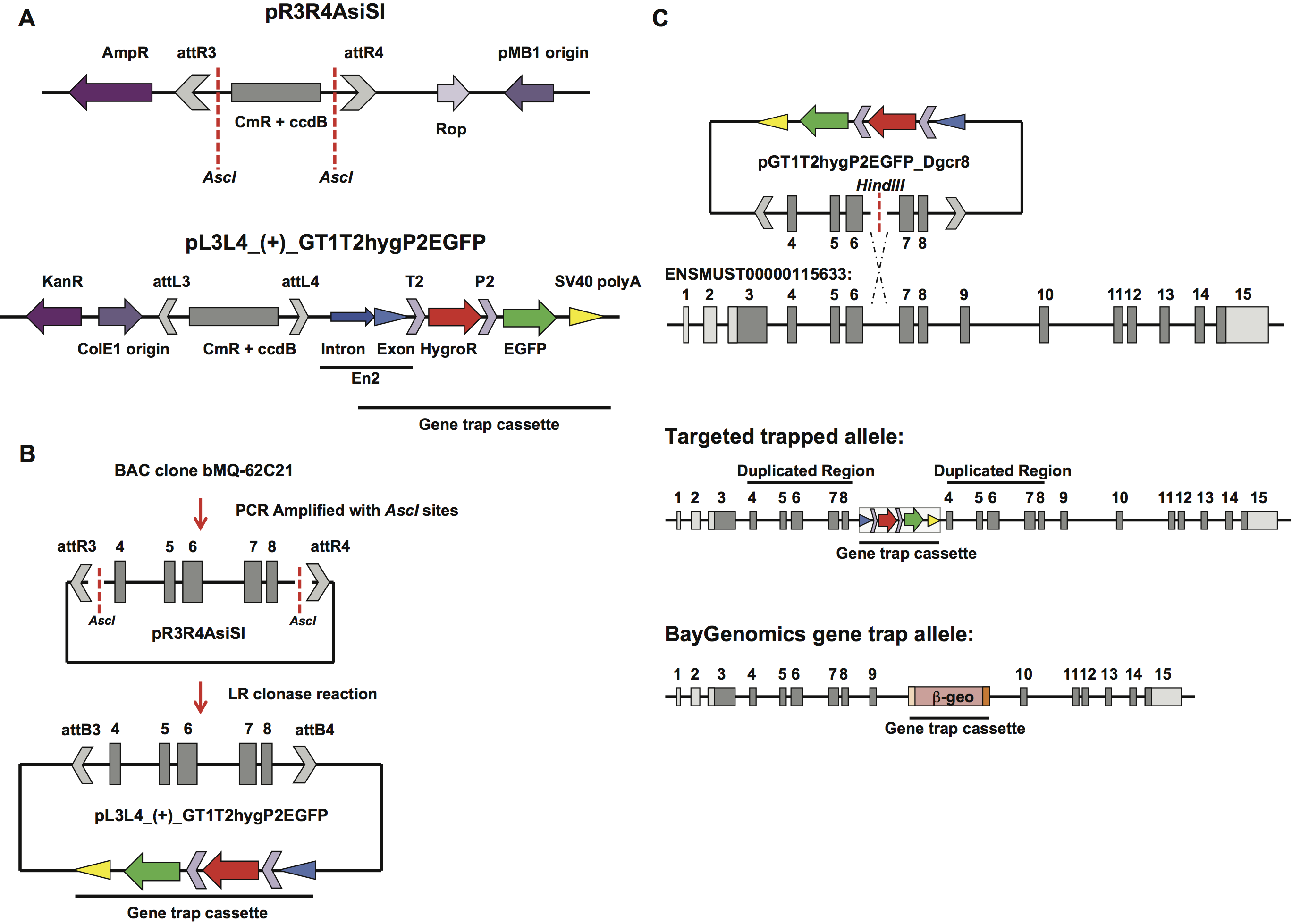

Supplement: Figure S1 — Schematic illustration of the gene targeting strategy used in this study. A) Map of vectors introduced in this study. The pR3R4AsiSI plasmid is a Gateway shuttle vector for cloning genomic DNA fragments. Positive and negative selection cassettes, CmR and ccdB, respectively are flanked by AscI restriction sites and attR3 and attR4 Gateway sites. The pL3L4_(+)_GT1T2hygP2EGFP plasmid contains a gene trapping cassette and attL3 and attL4 Gateway cloning sites to allow transfer of cloned genomic DNA fragments. The gene trap cassette is composed of the En2 splice acceptor, hygromycin resistance gene (HygroR), Enhanced Green Florescent protein gene (EGFP) and the SV40 polyadenylation site (SV40 polyA). The T2 and P2 sites cause ribosome skipping and are included for optimal expression of the resistance marker and fluorescent reporter [64]. B) Cloning of the homology region into the pL3L4_(+)_GT1T2hygP2EGFP vector. See Materials and Methods for a detailed description of the cloning strategy. The resulting targeting vector is named pGT1T2hygP2EGFP_Dgcr8. C) Schematic of the Dgcr8-targeted trapped and gene trapped alleles. The pGT1T2hygP2EGFP_Dgcr8 plasmid is an insertion-type gene-targeting vector containing exons 4 to 8 of Dgcr8. The vector is linearised within the homology region at a unique HindIII site prior to electroporation. The resulting targeted events cause a duplication of the homology region, placing the hygromycin-EGFP cassette downstream of exon 8. The BayGenomics gene trap cassette contains a β-geo reporter cassette, conferring G418 resistance and β-galactosidase activity, inserted downstream of exon 9. Insertion of the targeted cassette into the Bay Genomics gene trap allele will silence the β-geo reporter (Figure S2). (TIFF) [file pone.0041762.s001.tiff]

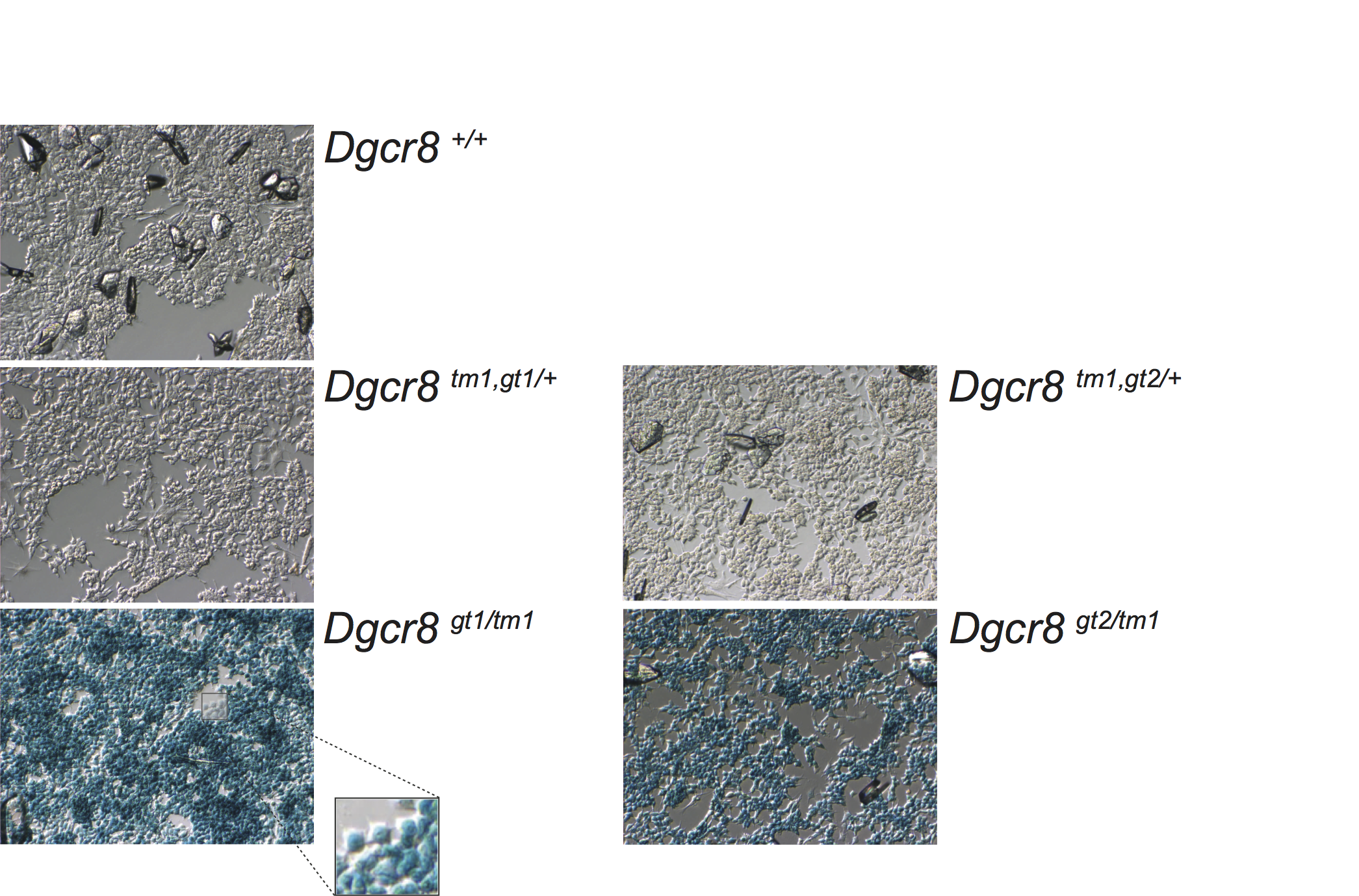

Supplement: Figure S2 — Xgal staining of cell lines to determine β-geo (β-galactosidase) activity associated with the initial gene trap. Xgal-staining confirmed that in the selected heterozygous cell lines (Dgcr8tm1,gt1/+ and Dgcr8tm1,gt2/+), the insertion of the second gene trap had disrupted the expression of the fusion transcript containing the original downstream construct and silenced the β-galactosidase activity of the fusion protein produced. This staining demonstrated that both gene traps are inserted within the same allele of the target gene. In contrast the homozygous mutant cell lines retained positive x-gal staining confirming that the gene traps must be within separate alleles of the gene. The inserted pane shows the nuclear localisation of the β-galactosidase activity of the β-geo fusion protein. (TIFF) [file pone.0041762.s002.tiff]

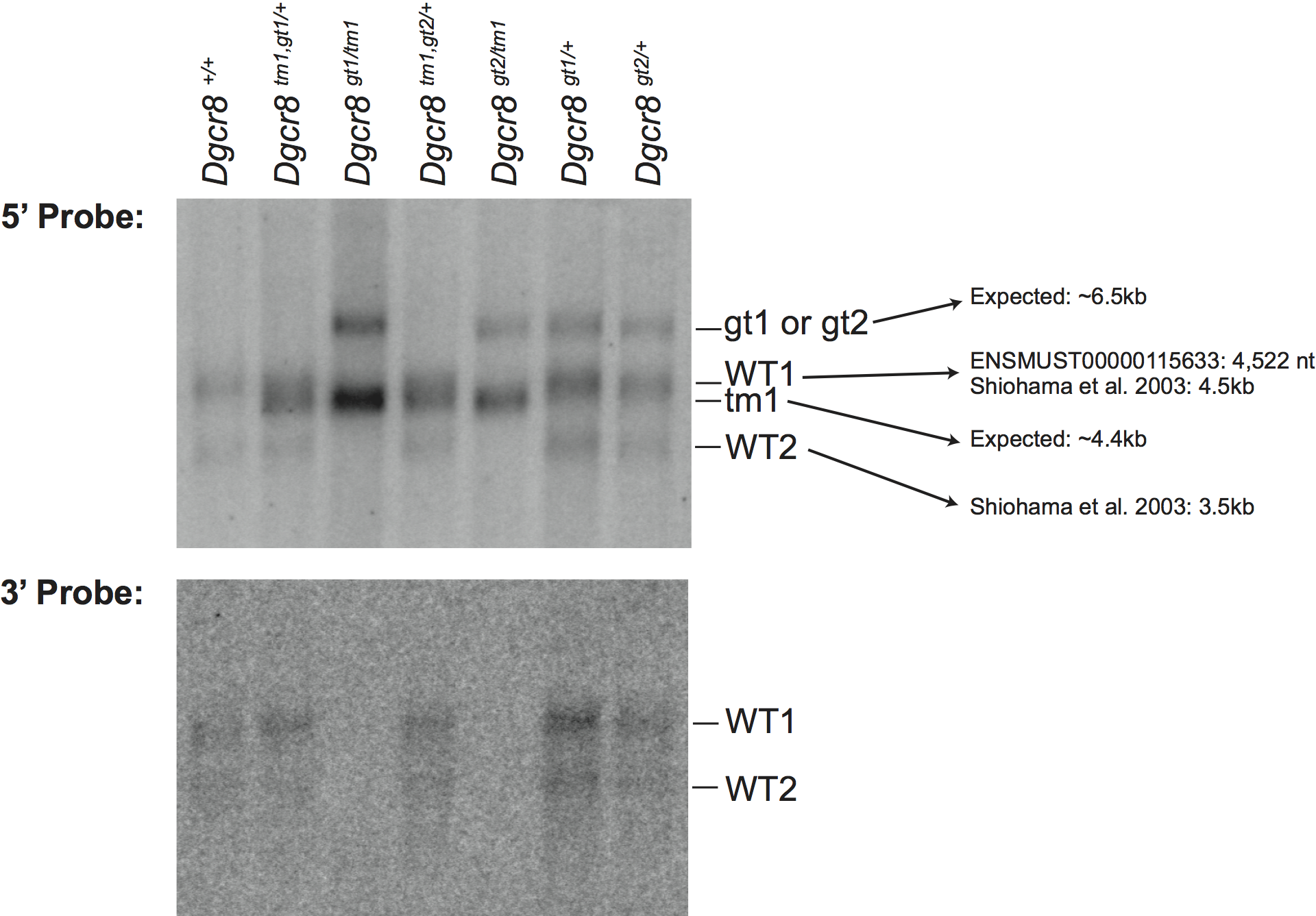

Supplement: Figure S3 — RNA blot of Dgcr8 derived transcripts assessing the expression of wild type and fusion transcripts. RNA derived from the two heterozygous cell lines were separated (Dgcr8tm1,gt1/+ and Dgcr8tm1,gt2/+) alongside the 2 homozygous mutants (Dgcr8gt1/tm1 and Dgcr8gt2/tm1) and the wild type cell line (Dgcr8 +/+). Additionally, RNA samples from the parental gene trap cell lines were also blotted (Dgcr8gt1/+ and Dgcr8gt2/+). The blot was hybridised sequentially with radiolabelled probes that either anneal to the 5′ (top) or 3′ (bottom) ends of the Dgcr8 transcripts. The expected transcript sizes are shown to the right of the blot. Size estimates for the gene-trapped transcripts are based on ENSMUST00000115633 and include the gene trap cassettes up to the polyadenylation sites. The nature of the smaller wild type transcript is unclear although the second small wild type transcript has previously been observed in mouse [65]. (TIFF) [file pone.0041762.s003.tiff]

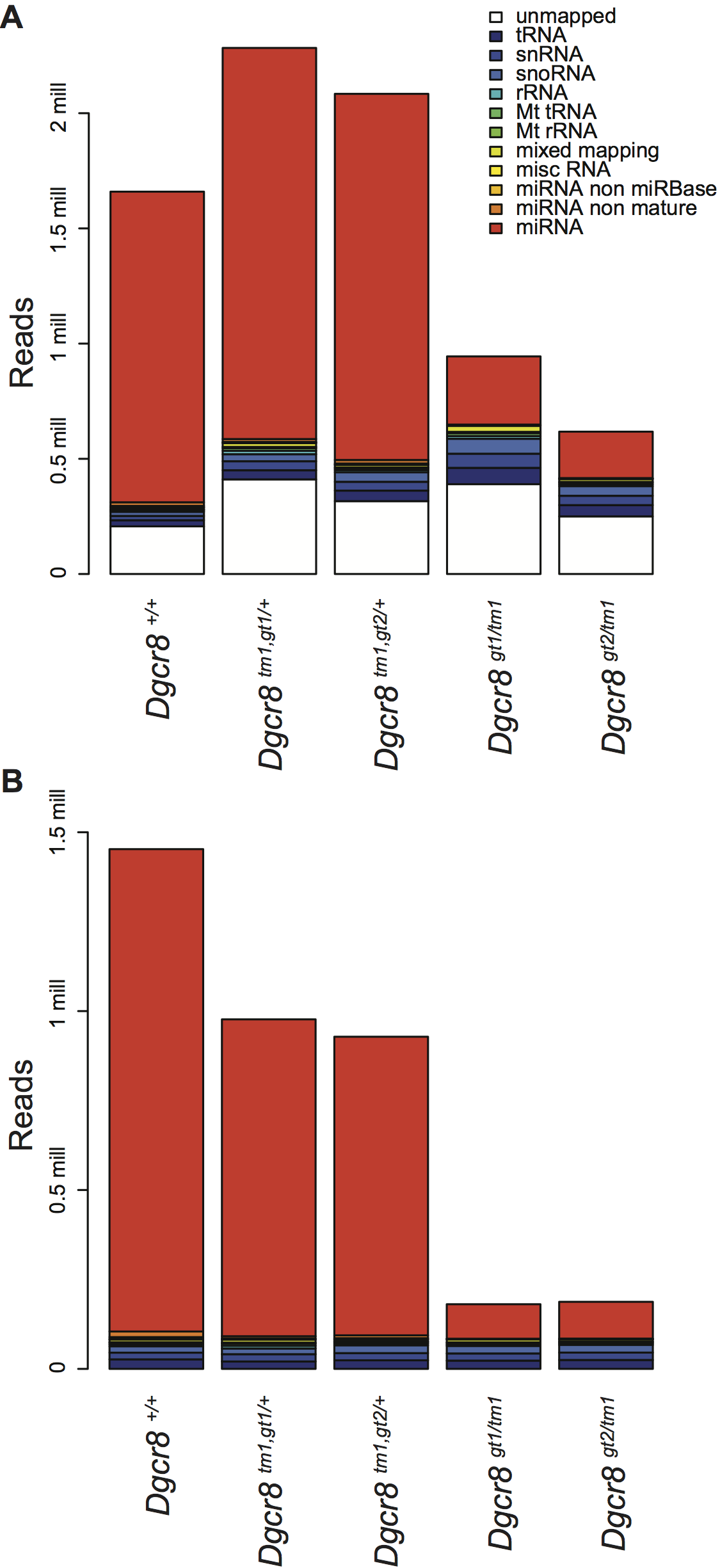

Supplement: Figure S4 — Read counts for each class of ncRNA in each cell line. A) Raw small RNA mapped read counts. B) Equivalent read counts after scaling to the non-miRNA non-coding RNA population in the WT sample. (TIFF) [file pone.0041762.s004.tiff]

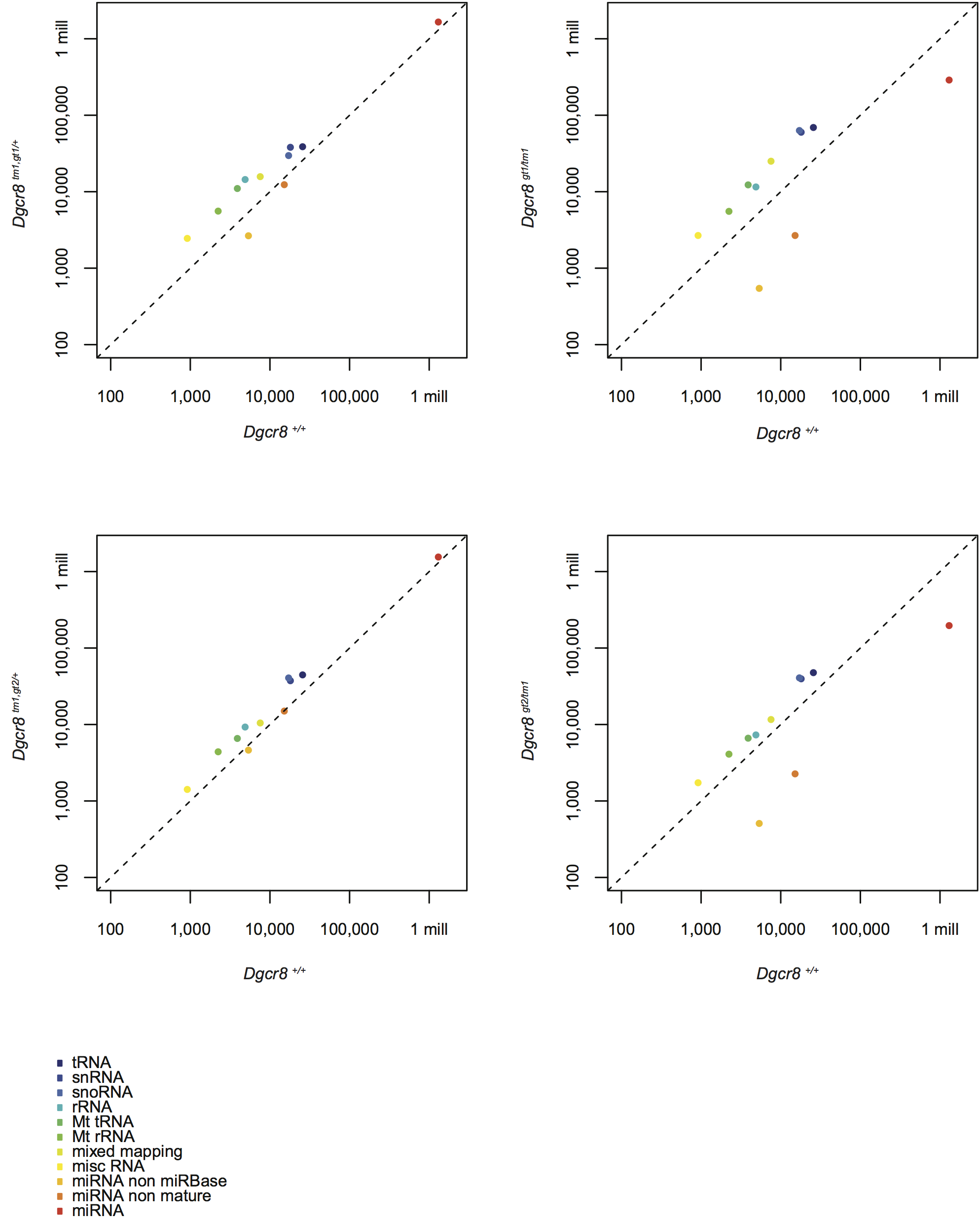

Supplement: Figure S5 — Total read counts for each RNA species compared between cell lines pre-normalisation. (TIFF) [file pone.0041762.s005.tiff]

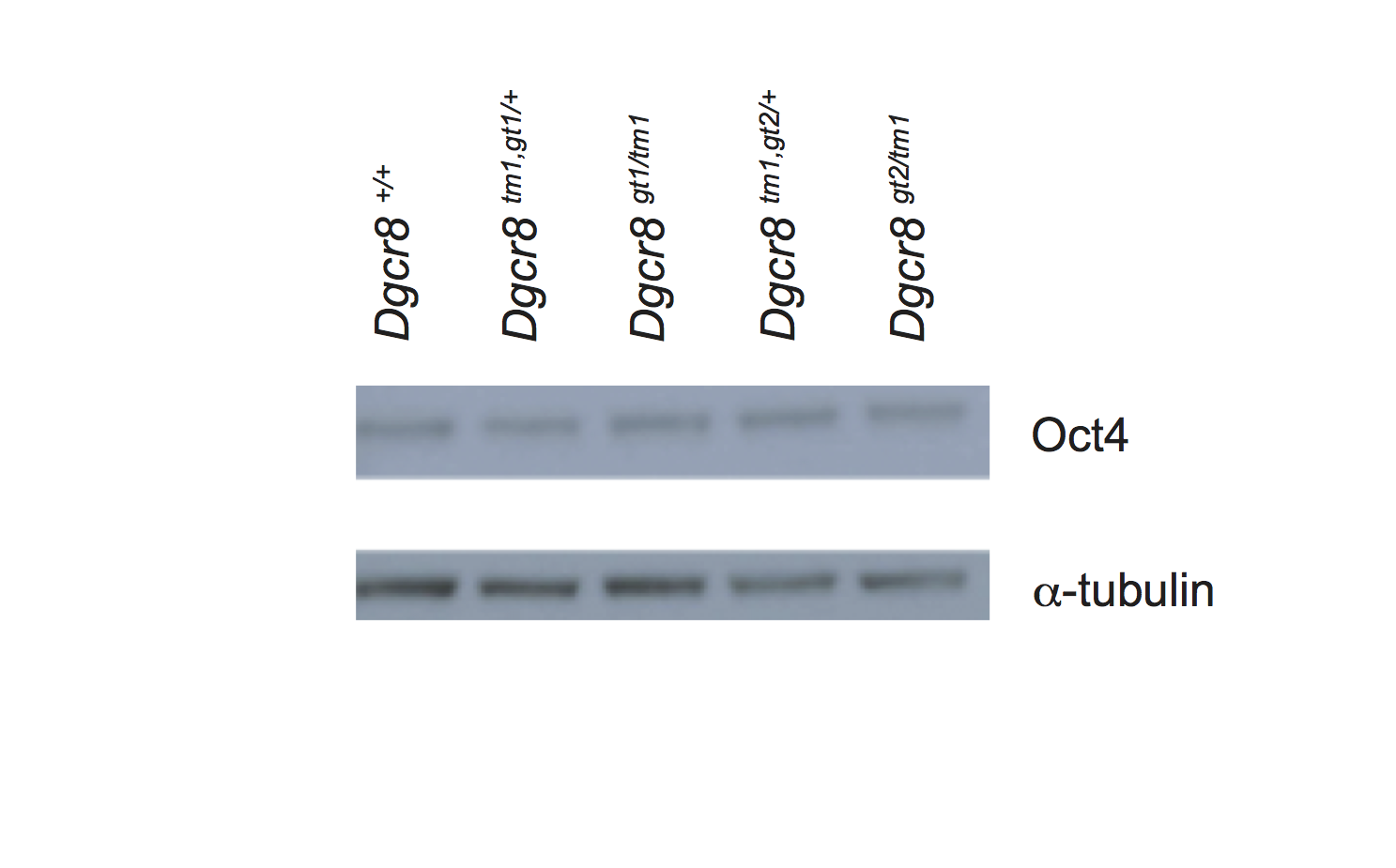

Supplement: Figure S6 — Western blot comparing the expression of Oct4 in Dgcr8 -depleted, heterozygous and wild type cell lines. The same blot was treated with antibodies for both Oct4 and α-tubulin (loading control). (TIFF) [file pone.0041762.s006.tiff]

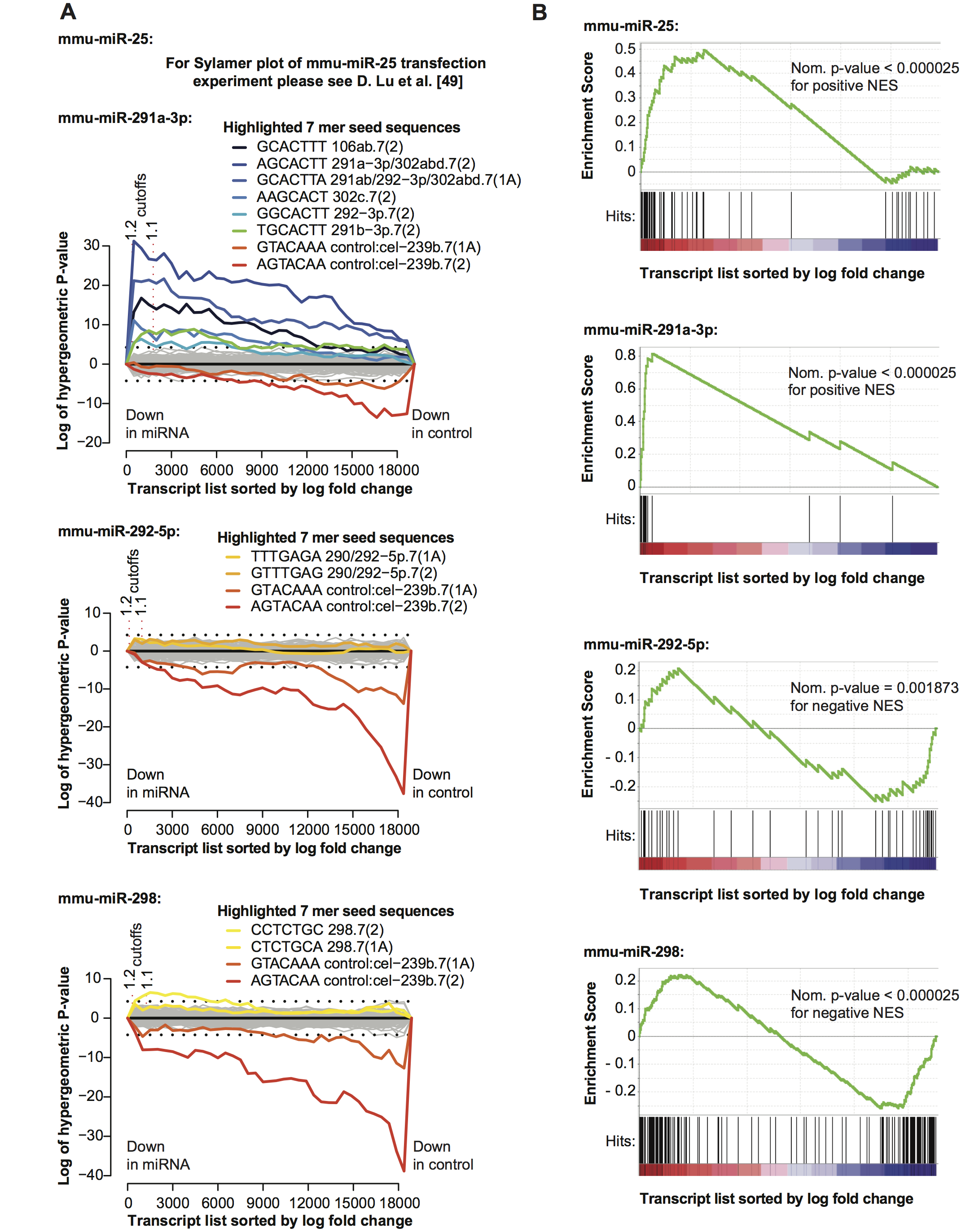

Supplement: Figure S7 — Global expression profiles to determine miRNA-dependent transcriptional effects. A: Sylamer plots comparing the expression profiles of Dgcr8 gt1/tm1 cells transfected with a miRNA mimic (miR-25, miR-291a-3p, miR-292-5p or miR-298) and a cel-miR-239b control miRNA. For a full description see Figure 3. B: GSEA enrichment plots [29] judging the enrichment of the transcripts within the miRNA target lists for miR-25, miR-291a-3p, miR-292-5p or miR-298 within regions of a list of transcripts ordered according to log fold change following the depletion of Dgcr8 in homozygous mutant cell lines. For a full description see Figure 3. (TIFF) [file pone.0041762.s007.tiff]

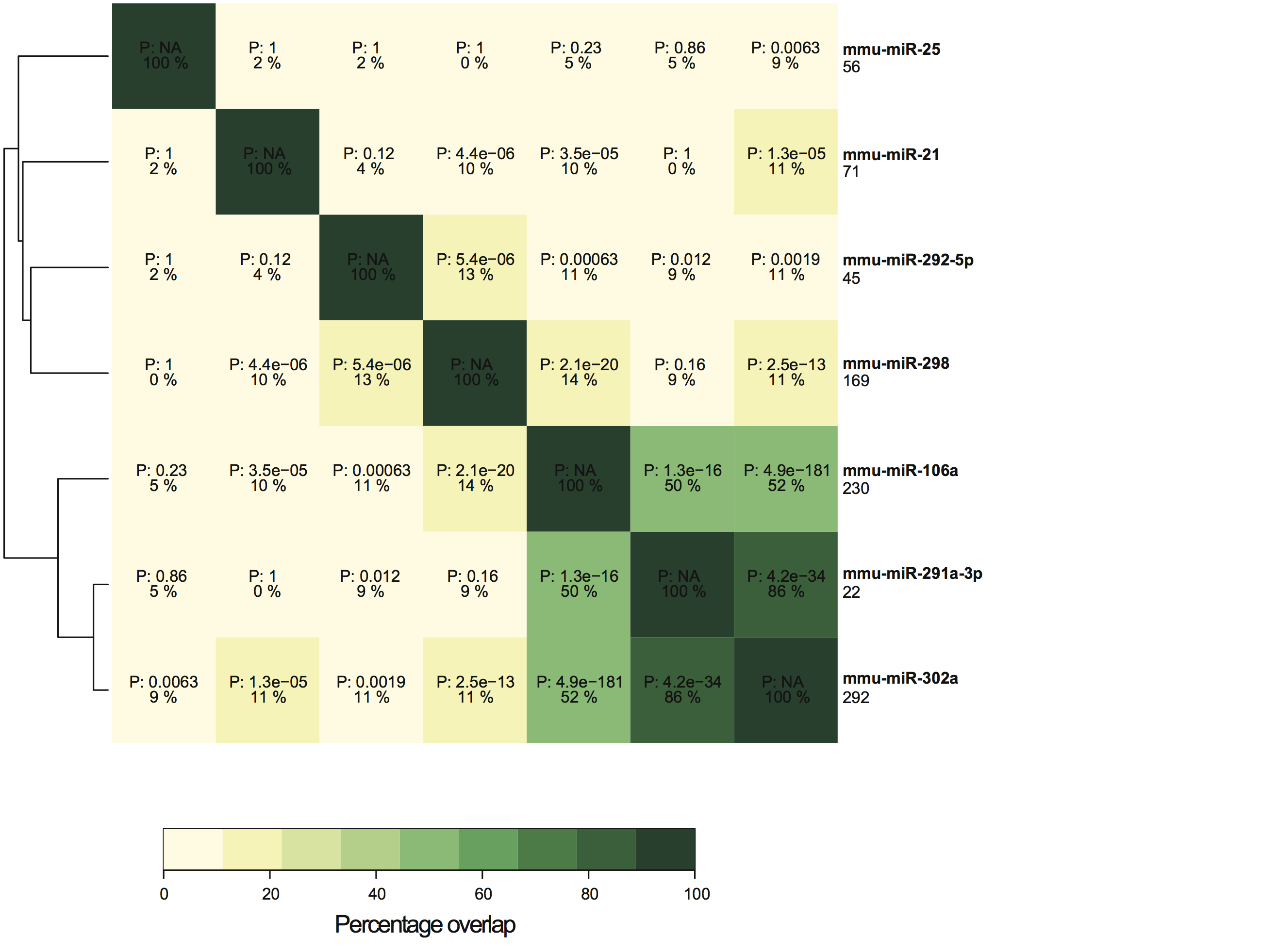

Supplement: Figure S8 — Overlap in target transcripts between each of the miRNAs. The percentage overlap represents the proportion of the transcripts within the smallest target set which overlap the larger set in each pairwise comparison between miRNA target sets. Bonferroni corrected hypergeometric P-values were calculated for each overlap. The universe consisted of those transcripts interrogated in the differential expression analyses. The number of potential targets identified for each miRNA is recorded beneath the miRNA name. In cases where targets were derived from alternative microarray versions, only transcripts interrogated by both platforms were considered in the comparison. (TIFF) [file pone.0041762.s008.tiff]

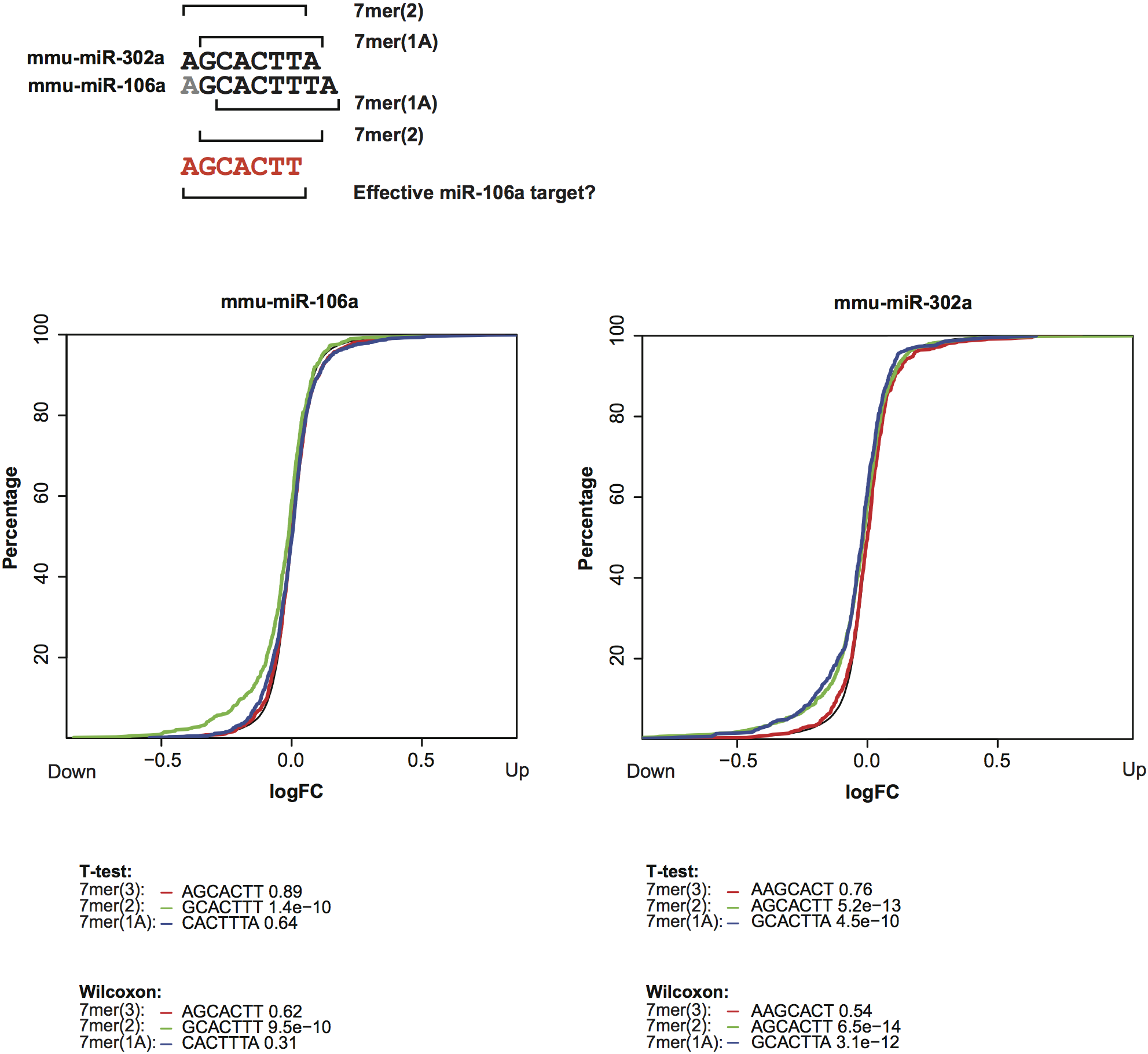

Supplement: Figure S9 — Assessing the potential for functional redundancy between miR-106a and miR-302a through a shifted 7mer(3) seed. A cumulative graph is presented of the relative effect of each miRNA seed sequence (7mer (1A), 7mer (2) and 7mer (3)) on transcripts which contain at least one seed from the relevant category and for which the seed is not part of a longer seed matching site (achieved through the exclusion of transcripts containing adjacent 7mers) (Red, green and blue lines) when compared to a 1/10 sampling of all transcripts represented on the array (Black), following the transfection of miR-302a or miR-106a miRNA mimics. P-values displayed were calculated using a Wilcoxon or T-test to determine if the relative distribution of the seed bearing transcripts, according to log fold change (logFC), differ significantly from the bulk of the other transcripts following miRNA transfection. The x-axis represents the relative logFC following miRNA transfection when compared to the cel-miR-239b. The y-axis is the cumulative percentage of each target set or transcripts represented on the array. (TIFF) [file pone.0041762.s009.tiff]

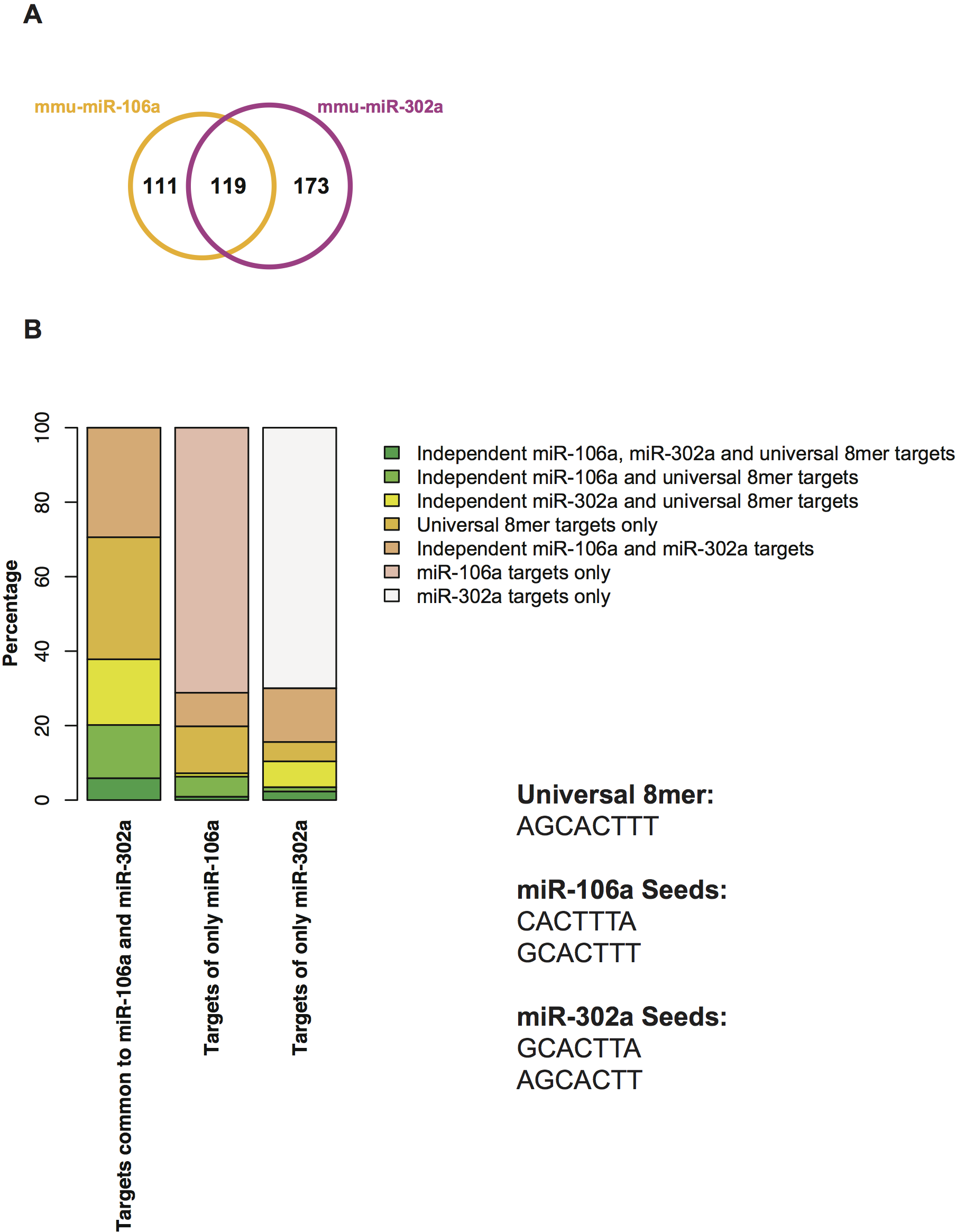

Supplement: Figure S10 — Investigation of the seed sequences found in miR-106a and miR-302a targets. A) A Venn diagram of transcripts within the target lists of both miR-106a and miR-302a. Shown are the numbers of transcripts found exclusively within the miR-302a target list and the miR-106a target list in addition to those shared by both. B) The class of seed sequence found in the 3′ UTRs of target transcripts. The nature of the miR-106a and miR-302a seed sequences found within the 3′ UTRs of the transcripts found exclusively in the miR-106a target list, the miR-302a target list or those transcripts within both target lists. The extended 8mer refers to the target sequence AGCACTTT, which is complementary to the accepted seed sequences of both miR-302a and miR-106a. The miR-302a and miR-106a target sites exclusively refer to those target sites that do not overlap these extended sites and are therefore not expected to be targeted by both miRNAs. (TIFF) [file pone.0041762.s010.tiff]

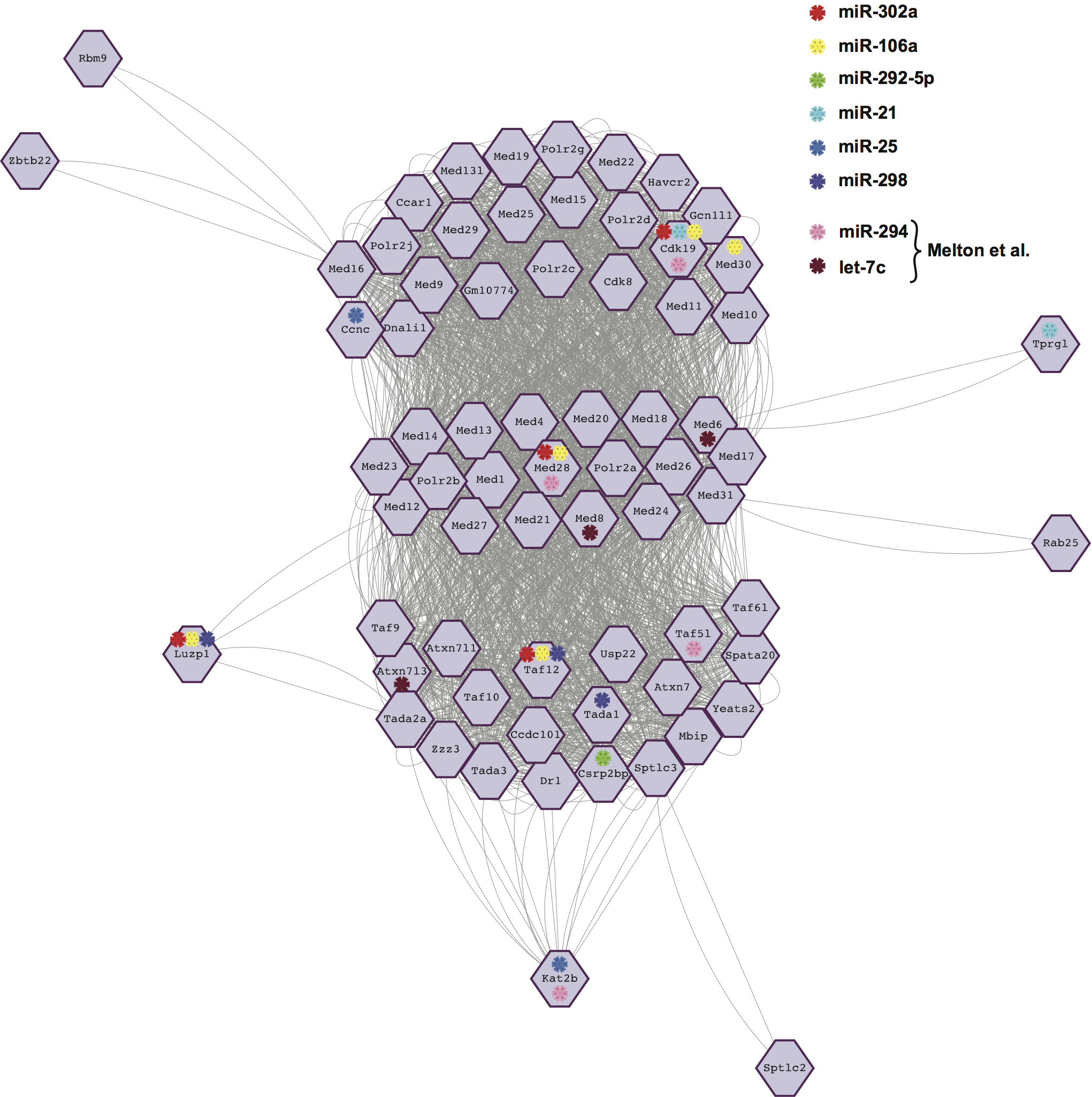

Supplement: Figure S11 — miRNAs found to target transcripts of proteins found within cluster 12 of the interaction network. Hexagons represent proteins and grey lines represent known interactions. Stars mark those genes in the sub-network targeted by miRNAs either in this study or in the work of Melton et al. [40] (see Materials and Methods). (TIFF) [file pone.0041762.s011.tiff]

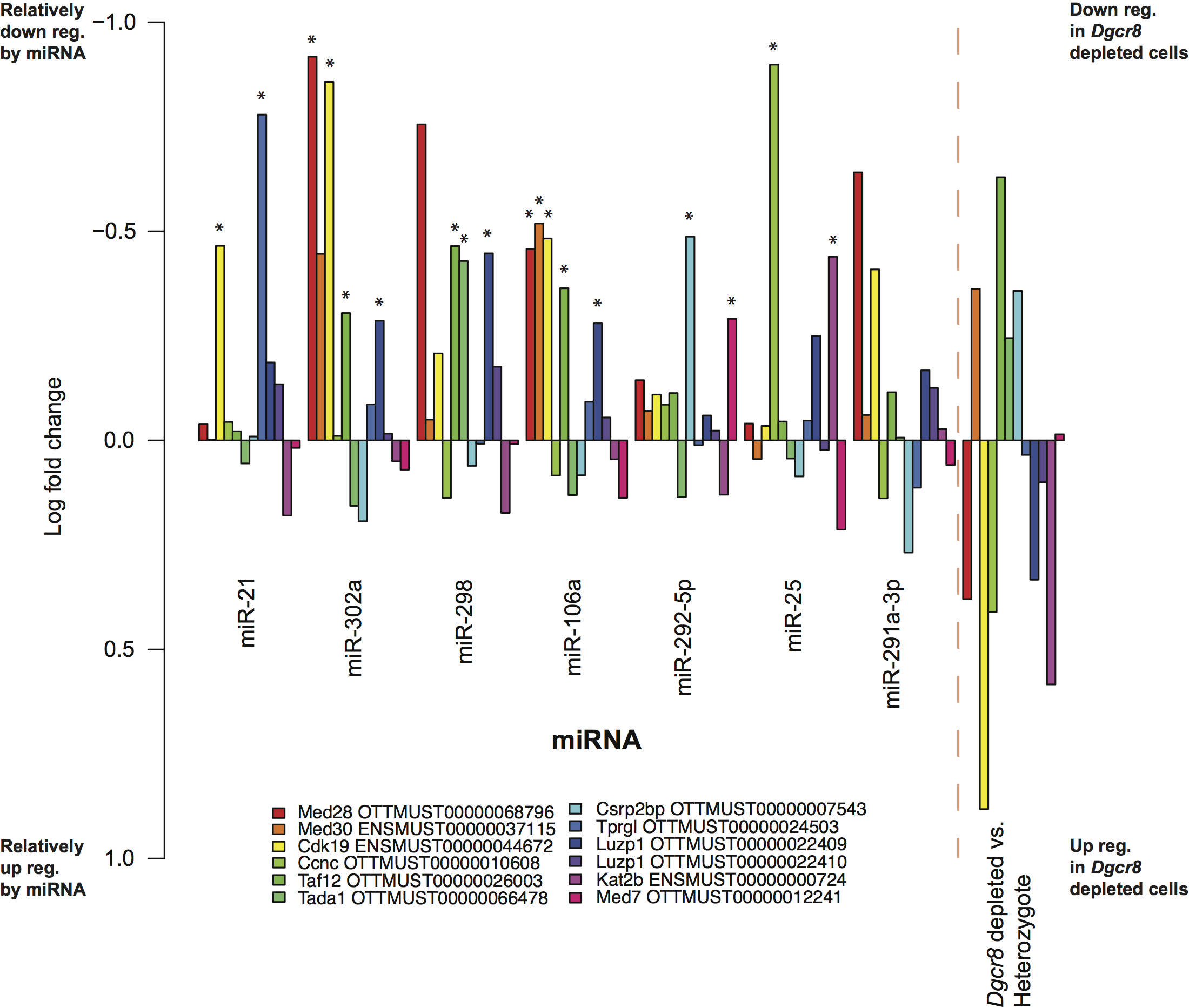

Supplement: Figure S12 — Relative disruption of miRNA targets within mediator associated cluster. Log fold change of transcripts associated with the miRNA targets from within cluster 12 (and Med7, a mediator protein missing from the cluster) upon the addition of each miRNA relative to the control miRNA (Left) and the log fold change upon the depletion of all miRNAs (Right). Stars represent those transcripts selected as potential targets of the transfected miRNA. (TIFF) [file pone.0041762.s012.tiff]
